# Gut Dysbiosis and Adaptive Immune Response in Diet-induced Obesity vs. Systemic Inflammation

**DOI:** 10.3389/fmicb.2017.01157

**Published:** 2017-06-22

**Authors:** Jana Pindjakova, Claudio Sartini, Oriana Lo Re, Francesca Rappa, Berengere Coupe, Benjamin Lelouvier, Valerio Pazienza, Manlio Vinciguerra

**Affiliations:** ^1^Center for Translational Medicine, International Clinical Research Center, St. Anne's University HospitalBrno, Czechia; ^2^Department of Primary Care and Population Health, University College LondonLondon, United Kingdom; ^3^Section of Human Anatomy, Department of Experimental Biomedicine and Clinical Neurosciences, University of PalermoPalermo, Italy; ^4^VaiomerLabège, France; ^5^Gastroenterology Unit, IRCCS “Casa Sollievo della Sofferenza” HospitalSan Giovanni Rotondo, Italy; ^6^Division of Medicine, Institute for Liver and Digestive Health, University College LondonLondon, United Kingdom

**Keywords:** obesity, inflammation, gut microbiota, adaptive immune system

## Abstract

A mutual interplay exists between adaptive immune system and gut microbiota. Altered gut microbial ecosystems are associated with the metabolic syndrome, occurring in most obese individuals. However, it is unknown why 10–25% of obese individuals are metabolically healthy, while normal weight individuals can develop inflammation and atherosclerosis. We modeled these specific metabolic conditions in mice fed with a chow diet, an obesogenic but not inflammatory diet—mimicking healthy obesity, or Paigen diet—mimicking inflammation in the lean subjects. We analyzed a range of markers and cytokines in the aorta, heart, abdominal fat, liver and spleen, and metagenomics analyses were performed on stool samples. T lymphocytes infiltration was found in the aorta and in the liver upon both diets, however a significant increase in CD4+ and CD8+ cells was found only in the heart of Paigen-fed animals, paralleled by increased expression of IL-1, IL-4, IL-6, IL-17, and IFN-γ. Bacteroidia, Deltaproteobacteria, and Verrucomicrobia dominated in mice fed Paigen diet, while Gammaproteobacteria, Delataproteobacteria, and Erysipelotrichia were more abundant in obese mice. Mice reproducing human metabolic exceptions displayed gut microbiota phylogenetically distinct from normal diet-fed mice, and correlated with specific adaptive immune responses. Diet composition thus has a pervasive role in co-regulating adaptive immunity and the diversity of microbiota.

## Introduction

The main feature of obesity is an excess of adipose tissue, which is the result of an imbalance existing between the intake and the expenditure of energy. The causes of obesity are both genetic and environmental; the diseases often comes along with the establishment of several chronic co-morbidities, such as high fasting hyperglycaemia, hypertriglyceridemia, dyslipidaemia, and hypertension (Alberti et al., 2005). Clinical diagnosis of metabolic syndrome is defined by the co-presence of at least three of the above criteria (Alberti et al., 2005). Metabolic syndrome enhances the odds of having type 2 diabetes and of developing diseases of the cardiovascular system. The majority of people with the metabolic syndrome are in obese, suggesting that the excess mass of adipose tissue may play a causative role in this cluster of diseases (Despres et al., 2008). However, this hypothesis has been strongly debated because several epidemiological analyses have evidenced people with a normal body mass index (BMI) who nevertheless display markers of inflammation and metabolic diseases [here termed metabolic syndrome leans (MSL)], such as high levels of triglycerides and accumulation of fat in the liver (Alberti et al., 2005); in fact, independently of BMI, and with variability linked to race and geographical areas, approximately 1 adult in every 4 or 5 had metabolic syndrome (Alberti et al., 2005). Conversely, a lack of clinical consistency for several or all metabolic syndrome components is found in some individuals with long-established and morbid obesity, which is actually recognized as healthy despite a high BMI. These subjects are referred to as metabolically healthy obese (MHO), and their prevalence has been estimated to be between 10 and 40% of the obese population, notwithstanding design differences between studies, such as age, ethnicity, geography, sample size, and the lack of a standardization (Munoz-Garach et al., 2016). As the prevalence of obesity and metabolic syndrome rises continuously with enormous economic and social costs, innovative countermeasures on the biological mechanisms, beyond prevention and lifestyle interventions, are required. In particular, the biological and disease mechanisms underlying the pathology of MSL and the health of MHO are not understood. Inflammation has been persistently associated with both obesity-associated diseases and the metabolic syndrome, indicating that low-grade inflammation is a potential and modifiable risk factor (Cox et al., 2015). The gut microbiota can be considered a distinct organ with endocrine properties; gut microbiota is involved, through a tight molecular interplay with the host organism, in the homeostasis of host organism energy and in stimulating of its immune system (Clarke et al., 2014). It has been proposed that gut microbiota participates to the establishment of metabolic diseases via the onset of low-grade inflammatory processes (Zupancic et al., 2012; Marchesi et al., 2016), and its composition is rapidly and heavily modulated by the diet (David et al., 2014). However, under healthy conditions commensal bacteria colonizing the gut interplay with the host immunity to maintain a state of homeostasis. In this respect, an immune system–gut microbiota cooperation which operates at optimal levels is instrumental for setting protective mechanisms against pathogenic agents and, at the same time, for keeping in check the regulatory pathways implicated in the avoidance of triggering immune responses to harmless antigens (Belkaid and Hand, 2014). This reciprocal interaction involves both innate (Thaiss et al., 2014) and adaptive immunity (Kato et al., 2014; Zhang and Luo, 2015). In this respect, signals from gut microbiome play crucial role in maturation (or differentiation) of IL-17 expressing Th17 cells as well as IFN-γ expressing Th1 cells (Ivanov et al., 2008; Gaboriau-Routhiau et al., 2009). Although, it has been suggested that dysbiosis can cause immune dysfunctions by activating B and T cells regardless of their distance from the location of their induction (Honda and Littman, 2016), there is scarce knowledge on the relationship between distinct immune cell populations, more in particular those belonging to the adaptive immunity, and the heterogeneity of digestive system-residing and symbiotic bacteria.

Here we modeled the metabolic and clinical features of MSL and MHO humans in C57/BL6 mice fed for 20 weeks with a chow diet, a high fat obesogenic but not inflammatory diet (mimicking healthy obesity) or a hypercholesteraemic, pro-atherogenic, low fat diet (Paigen diet, mimicking systemic inflammation, and fatty liver in the lean subjects; Getz and Reardon, 2006), under the same housing environment. We then analyzed possible interactions among adaptive immune system in multiple tissues, and gut microbiota. Mice fed these distinct “unhealthy” diets reproducing human metabolic exceptions, MSL and MHO, had a gut microbiota with phylogenetic characteristics significantly divergent from normal diet-fed littermates, and displayed specific intra-tissue adaptive immune responses.

## Materials and methods

### Dietary mice models

Four week old male C57BL/6 (B6) mice were purchased from Velaz, Ltd. (Prague, Czech Republic). Animals (*n* = 10 per experimental group) were housed in specific pathogen-free facilities and fed *ad libitum* with basal (normal) or specific (Paigen or Western) diet for 20 weeks with fresh, clean water available at all time. Diet compositions were as following: normal diet (ND): proteins % 18.6, fat % 10 (Linoleic Acid, % 3.34, Linolenic Acid, % 0.07 Arachidonic Acid, % 0.01, Omega-3 FA, % 0.07, Saturated FA % 2.72, Monounsaturated FA, % 3.31, Polyunsaturated FA, % 3.42), carbohydrates % 60.6, cholesterol % 0, choline chloride % 0; High fat diet (HD): proteins % 17.3, fat % 21.2 (Linoleic Acid, % 1.70, Linolenic Acid, % 0.16 Arachidonic Acid, % 0.02 Omega-3 FA, % 0.23, Saturated FA, % 7.92 Monounsaturated FA, 6.28, Polyunsaturated FA, % 3.14), carbohydrate % 48.5, cholesterol % 0, choline chloride % 0.2); Paigen diet (PD): proteins % 20.8, fat % 15 (Linoleic Acid, % 1.70, Linolenic Acid, % 0.14, Arachidonic Acid, % 0.01, Omega-3 FA, % 0.16, Saturated FA % 7.18 Monounsaturated FA, % 5.34, Polyunsaturated FA, % 1.91), carbohydrate % 61, cholesterol % 1.25, choline chloride % 0.5. The experiments were performed in accordance with the law governing the protection of animals and the principles derived from the requirements of the Act No. 359/2012 Sb., on the protection of animals against cruelty and the decree 419/2012 Sb. Ministry of Agriculture of Czech Republic on the protection of experimental animals (including relevant EU regulations). The experiments were approved by the local Animal Ethics Committee on the Welfare of Experimental Animals and by the Ministry of Education of Czech Republic (MSMT-2582/2016-14)—project number 66-2015. Serum levels of fasting glucose, fasting insulin, triglycerides, and cholesterol were measured as we have previously described (Cederroth et al., 2008; Pazienza et al., 2016).

### Histology

Samples of liver, aorta, and heart from each mouse and were fixed in formalin and embedded in paraffin for histological analysis. Sections with a thickness of 4 μm were obtained from paraffin blocks and stained with hematoxylin and eosin for histological examinations (Benegiamo et al., 2013). Histological classification of NAFLD was performed by applying a semiquantitative scoring system grouping histological traits into broad classes (steatosis, fibrosis, portal inflammation, hepatocellular injury, and miscellaneous features; Kleiner et al., 2005).

### Tissue digestion and single cell suspension preparation

To prepare single cell suspension from solid tissue (aorta, heart, and abdominal fat), required digestion, the tissue was minced with a sterile scissors and placed in 1 ml DMEM containing: for heart and aorta—2.5 mg/ml Collagenase type XI, 0.25 mg/ml Hyaluronidase type I-s, 0.25 mg/ml DNase I, 2.5 mg Collagenase type I; for abdominal fat −1 mg/ml Collagenase IV of 3% DMEM. Tissues were incubated in water bath for 1 h with vortex every 15 min and washed by cold PBS. Erythrocytes were removed by RBC lysis buffer (Biolegend), cells were washed by PBS and transferred to fresh tubes through 70 mm nylon mesh. Finally, the cell suspension was resuspended in 1 ml PBS per sample. Spleen and liver were cut into small pieces and passed through tissue grinder to Petri dish, and then the cell suspension was passed through the 70 μm cell strainers and processed as mentioned above. Peripheral blood was collected into heparinized syringe, resuspend in PBS and spin down. Erythrocytes were removed by RBC lysis buffer and cells passed through the 70 μm cell strainer. Single cell suspensions were used for flow cytometry or PCR.

### Flow cytometry

Cells in single cell suspensions were stained in 100 μl aliquots of FACS buffer (2% FBS in PBS) after incubation with fluorochrome-labeled antibodies for 30 min at 4°C followed by washing in FACS buffer. Combination of surface markers for T-lymphocytes was CD45, CD4, and CD8 and myeloid cell subsets were stained for CD45, CD11b, CD11c, F4/80, and Ly6G using specific antibodies (Biolegend). Analysis was performed using a BD Biosciences FACSCanto® flow cytometer and FlowJo® software (TreeStar Inc., Olten, Switzerland).

### Gene expression

Total RNA was isolated from cell suspensions using Trizol LS Reagent (Life Technologies). RNA was converted to cDNA using gb Reverse Transcription Kit (Generi-Biotech, Czech Republic). Equal amounts of cDNA were analyzed by Real-Time quantitative PCR using gb Elite PCR Master Mix (Generi-Biotech, Czech Republic) on a LightCycler® 480 Real Time PCR System (Roche). Relative quantifications were performed using the comparative CT method with normalization to GAPDH and results expressed as fold difference relative to a relevant control sample. Primers and probes were from Qiagen: GAPDH (Mm99999915_g1), IL-17A (Mm00439618_m1), IFN-γ (Mm01168134_m1), IL-4 (Mm00445259_m1), TGF-β (Mm01178820_m1), IL-6 (Mm00446190_m1), IL-12 p35 (Mm00434165_m1).

### Metagenomics profiling

The microbial population present in the fecal samples from mice was determined using next generation high throughput sequencing of variable regions of the 16S rRNA bacterial gene. The workflow performed at VAIOMER (France) includes the steps of (i) Library construction and sequencing; (ii) PCR amplification was performed using 16S universal primers targeting the V3–V4 region of the bacterial 16S ribosomal gene (Vaiomer universal 16S primers). The joint pair length was set to encompass 476 base pairs amplicon thanks to 2 × 300 paired-end MiSeq kit V3. For each sample, a sequencing library was generated by addition of sequencing adapters. The detection of the sequencing fragments was performed using MiSeq Illumina® technology; (iii) Bioinformatics pipeline, The targeted metagenomic sequences from microbiota were analyzed using the bioinformatics pipeline established by Vaiomer from the FROGS v1.3.0 guidelines. Briefly, after demultiplexing of the bar coded Illumina paired reads, single read sequences are cleaned and paired for each sample independently into longer fragments. Operational taxonomic units (OTU) are produced via single-linkage clustering and taxonomic assignment is performed in order to determine community profiles. PhyloSeq v1.14.0 R package was used to provide a set of classes and tools to facilitate the import, storage, analysis, and graphical display of microbiome census data. The samples with <5,000 sequences after FROGS processing were not included in the statistics (rarefaction analysis, alpha diversity, beta diversity-multidimensional scaling). The raw sequencing data are available upon request.

#### LEfSe method

The OTU files generated were uploaded and formatted for LEfSe analysis using the per sample normalization of sum values option. The linear discriminant analysis effect size was determined using default values (alpha value of 0.5 for both the factorial Kruskal–Wallis test among classes and the pairwise Wilcoxon test between subclasses, threshold of 2.0 for the logarithmic LDA score for discriminative features) and the strategy for multi-class analysis set to “allagainst-all.” LEfSe cladograms from the LDS effect size data were generated with Bacteria as the tree root. Differential features detected as biomarkers from the raw data used to generate the cladograms were plotted as abundance histograms with class and subclass information.

### Statistical methods

The parametric Student's *t*-test (2-sample *t*-test) was used to compare the difference in mean of immune cells by type of diet (HD vs. ND), and difference in mean of cytokines by type of diet (HD vs. ND). The non-parametric Mann–Whitney *U*-test was also used to check if the results were basically similar to the *t*-test using GraphPad Prism Software (version 5.00 for Windows, San Diego, CA, USA): a *p* < 0.05 was considered significant. To explore the association of gut microbiota with immune cells and cytokines levels, analyses were carried out using STATA/SE software. As preliminary analysis, mean and standard deviation (SD) of each gut microbiota type and adaptive immune system parameters measured in the aorta, heart, liver, spleen, and fat were calculated. The Pearson's correlations between each gut microbiota and adaptive immune system parameters were also examined. In the final analysis, associations between gut microbiota and adaptive immune system parameters levels were explored by using linear regression models. In each of the models, the associations between each bacterial taxa and adaptive immune system parameters were reported as absolute difference (β), with 95% Confidence Intervals (CI), in immune system parameters levels by % of increase in the proportion of the bacterial taxa. Coefficient of determination (*R*^2^) was also reported.

## Results

### Modeling healthy obesity and metabolic syndrome during leanness in mice

**Figure 1:**
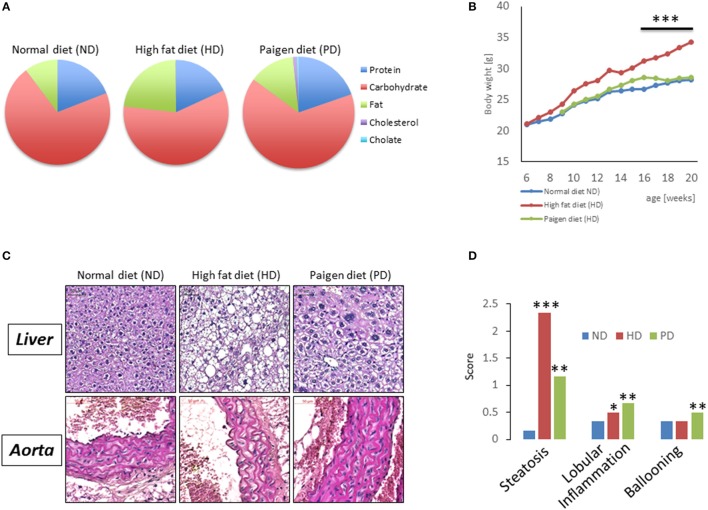
**(A)** Pie chart representing diet compositions in terms of fat, carbohydrates, choline, cholesterol, proteins. **(B)** Body weight of C57/BL6 mice fed for 20 weeks with a normal chow diet (ND), high fat diet (HD), or Paigen diet (PD). **(C)** Representative pictures from hematoxylin and eosin staining of liver sections (upper panels) and aorta sections (lower panels) in C57/BL6 mice fed with ND, HD, or PD. **(D)** Steatosis, lobular inflammation, and ballooning were scored semi quantitatively (0–4). ^*^p < 0.05; ^**^p < 0.01; ^***^p < 0.001 vs. ND.

**Figure 2:**
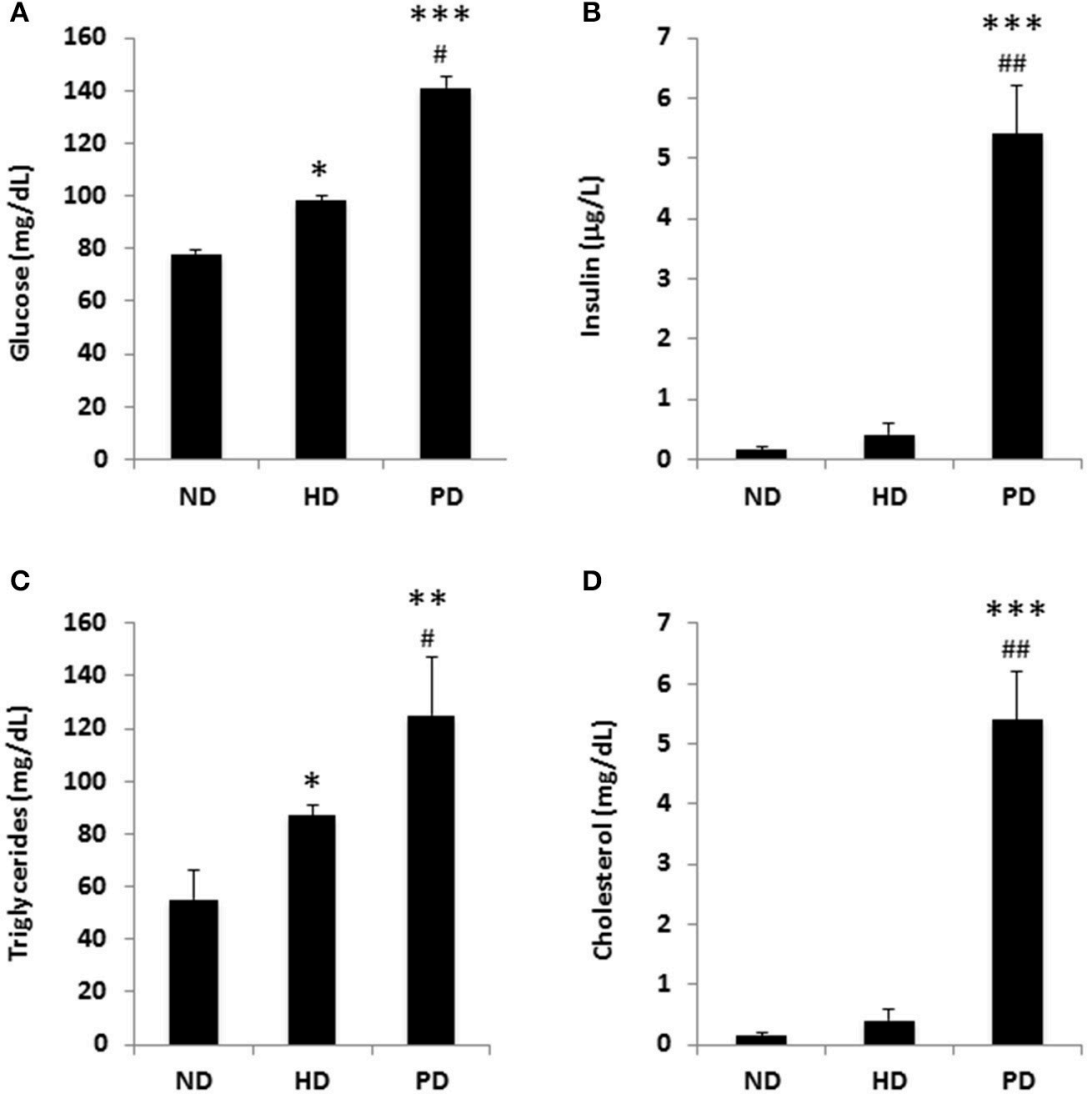
Metabolic parameters of C57BL/6 mice fed a standard normal chow (ND) or a high fat diet (HD) or a Paigen diet (PD) for 15 weeks. **(A)** fasting glucose; **(B)** fasting insulin; **(C)** serum triglycerides; **(D)** serum cholesterol. *N* = 4. ^*^p < 0.05, ^**^p < 0.01, ^***^p < 0.001 vs. ND; ^#^*p* < 0.05, ^##^*p* < 0.01 vs. HD.

To model diets able to mimic MSL and MHO conditions in humans, three groups (*N* = 10) of 4 weeks old C57/BL6 mice were fed different dietary regimens: (i) a control normal diet (ND, 21.2% kcal from proteins, 58% kcal from carbohydrate, and 17% from fat); (ii) high fat diet, rich in fatty acids (HD, 21.2% kcal from proteins, 24% kcal from carbohydrate, and 58% from fat) and with 0.1% cholesterol, and (iii) atherogenic/inflammatory Paigen diet (PD), containing similar composition of the normal diet with in addition 1.25% cholesterol and 0.5% sodium cholate (Figure 1A). C57/BL6 mice had similar baseline weight before starting being fed the diets (mean = ~21 ± 0.4 g). After 15 weeks of dietary regimens, body weight was unchanged in mice on the control ND or the PD, which both increased body weight during growth by ~33% (ND = 28.3 ± 0.7 g and PD = 28.8 ± 0.77 g, respectively, Figure 1B). In contrast, mice on the HD gained ~65% in weight (HD = 35.9 ± 0.6 g), compared to their baseline, indicating that only HD diet was obesogenic (*p* < 0.001 vs. ND and vs. PD). We then examined glucose and insulin levels at the end of the dietary treatment. Basal insulin and glucose fasting levels were considerably higher in PD vs. ND and HD (Figures 2A,B). A similar trend was observed for serum triglycerides and cholesterol levels, which were highest in the PD group vs. ND and HD (Figures 2C,D). Obesogenic HD regimen triggered lipid accumulation in the liver under the form of simple steatosis, whereas atherogenic/inflammatory PD regimen induced NAFLD/NASH at the end of its pathologic spectrum, characterized by lipid accumulation, ballooning, fibrosis, and inflammatory infiltrates, as quantified by NAFLD/NASH score (Figure 1C upper panels, Figure 1D), consistent with previous finding that the cholesterol and cholate components of Paigen diet induces genes involved in inflammation and fibrosis, respectively, in the liver (Vergnes et al., 2003). Cross-sectional analysis of aortas walls suggested an increased infiltration of inflammatory cells in the PD-fed mice, in comparison to ND or HD fed mice (Figure 1C, lower panels). Altogether, these data indicate that PD triggers prominent features of metabolic syndrome and inflammation in mice in absence of weight gain, compared to obesogenic HD.

### Dissecting diet-dependent intra-tissue adaptive immune changes

**Figure 3:**
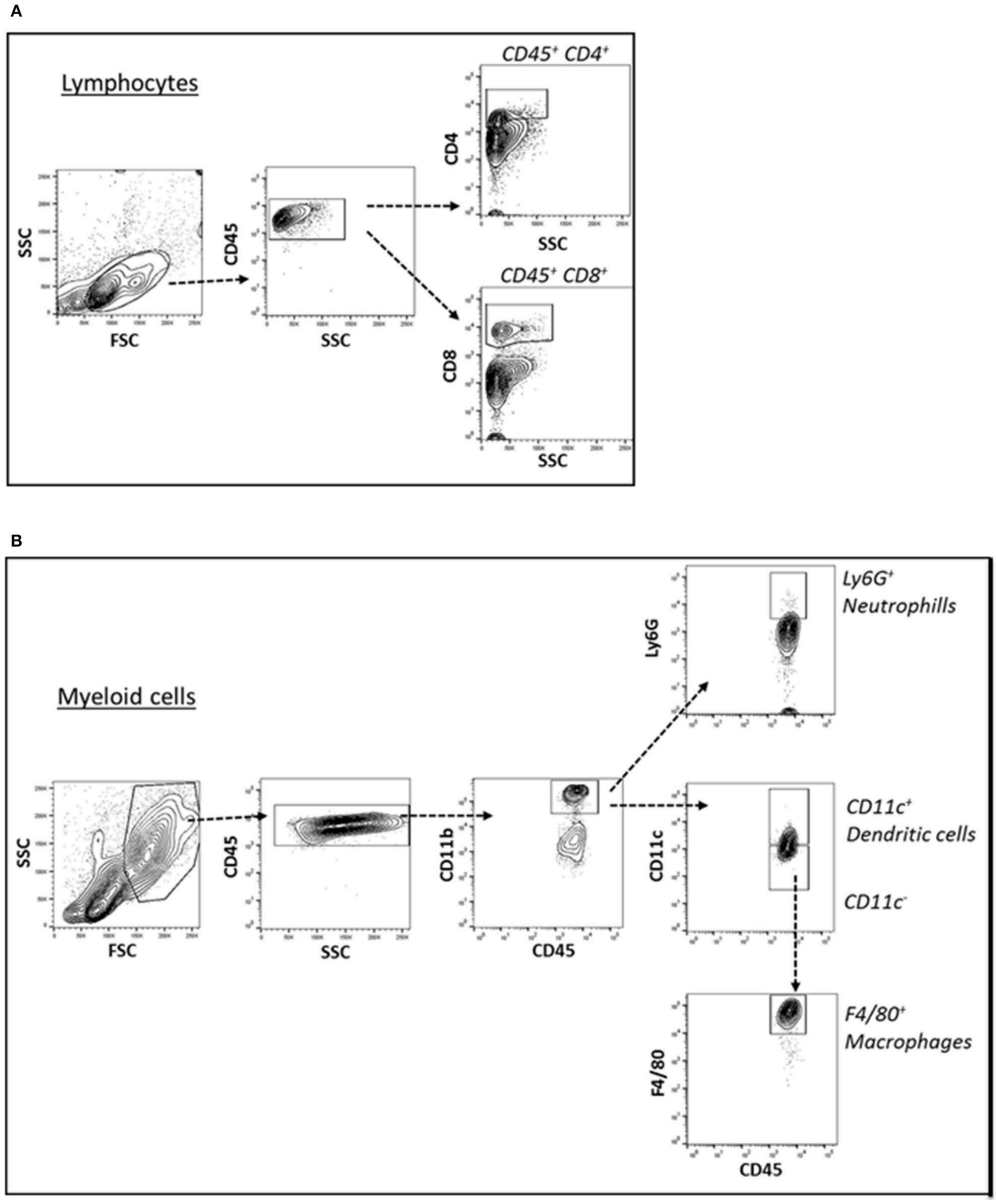
**(A)** Cell suspension obtained from tissues were surface-stained for T lymphocyte markers with antibody combination CD45, CD4, and CD8 **(A)** and gated for CD45+ CD4+ T lymphocytes and CD45+ CD8+ T lymphocytes. **(B)** For myeloid cell subsets, the cell suspensions were surface-stained with antibody against CD45, CD11b, CD11c, Ly6G, and F4/80 **(B)** and gated for CD45+ CD11b+ Ly6G+ neutrophils, CD45+ CD11b+ CD11c+ dendritic cells, CD45+ CD11b+ CD11c- F4/80+ macrophages.

**Figure 4:**
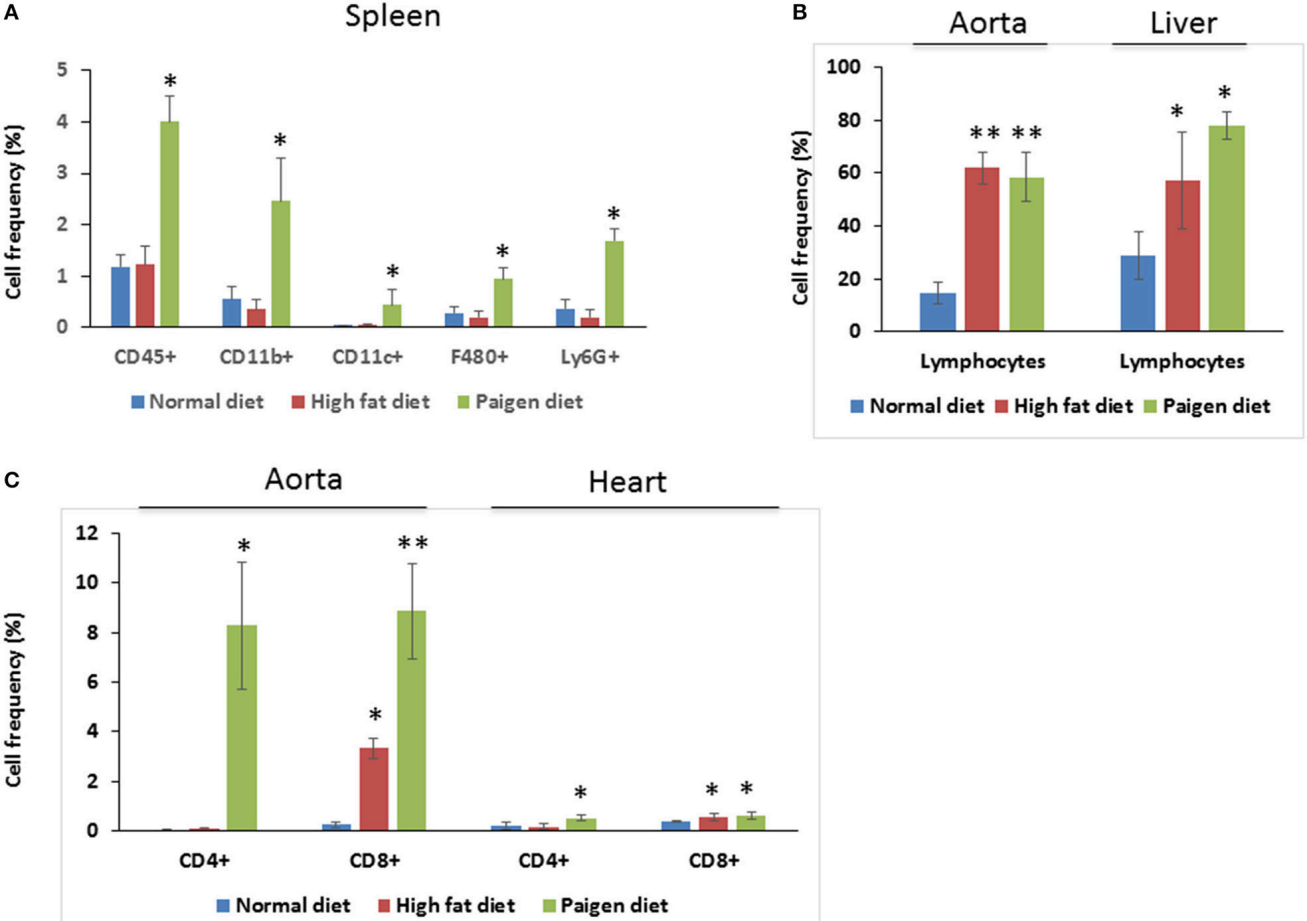
Immune cells profiling in the tissues of Normal diet (ND)-, High fat diet (HD)-, and Paigen diet (PD)-fed mice. Single cell suspensions were prepared from solid tissues (aorta, heart, and liver) and processed for FACS analyses. Combination of surface markers for T-lymphocytes was CD45, CD4, and CD8 and myeloid cells were stained for CD45, CD11b, CD11c, F4/80, and Ly6G. **(A)** Frequency of total myeloid cells and cells positive gated for CD45, CD11b, CD11c, F4/80, and Ly6G in the spleen. **(B)** Frequency of lymphocytes in the aorta and in the liver, of gated cells. **(C)** Frequency of cells gated for CD4 and CD8 in the aorta and in the heart. *N* = 3.4. ^*^p < 0.05; ^**^p < 0.01.

**Figure 5:**
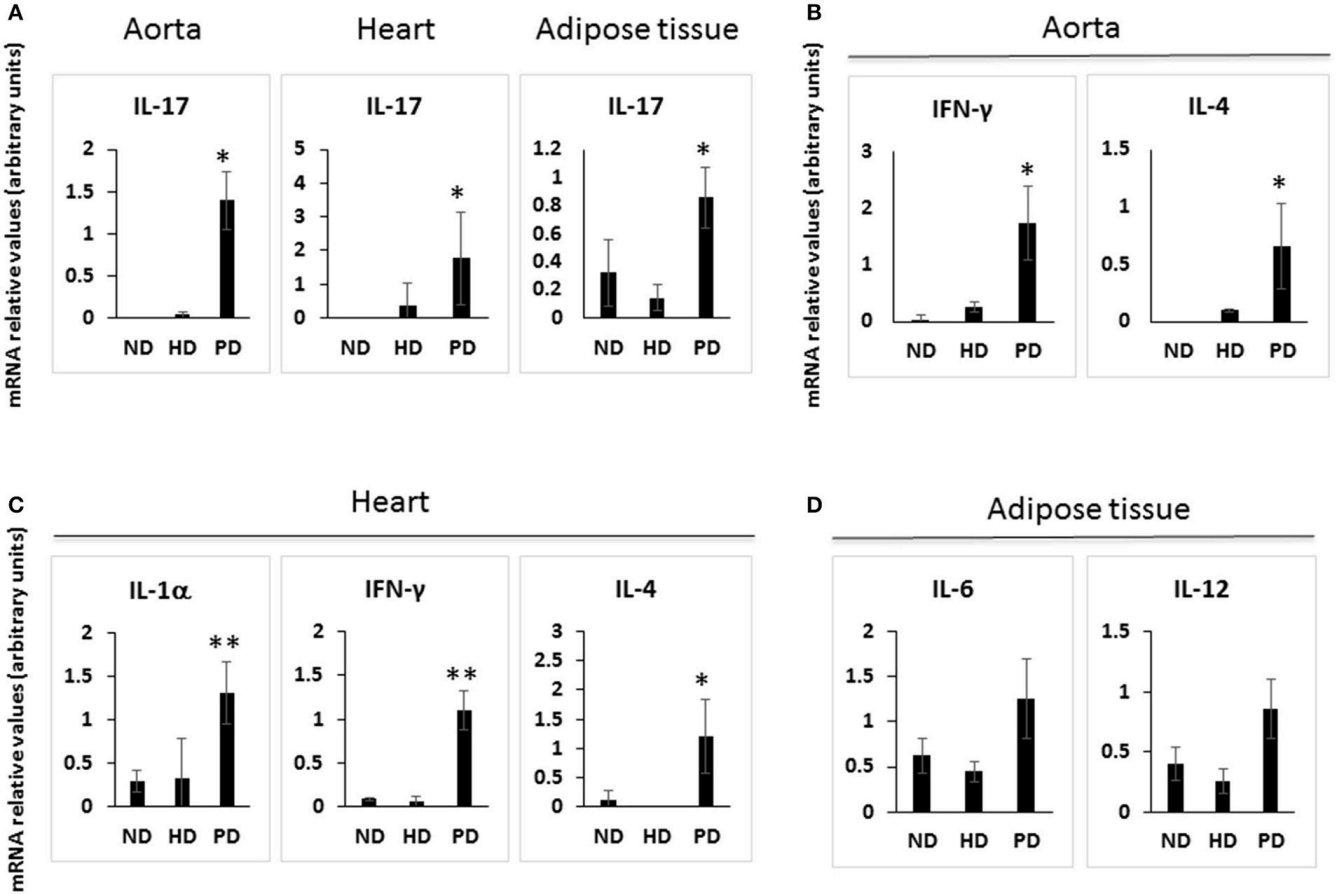
Cytokine gene expression in the tissues of Normal diet (ND)-, High fat diet (HD)-, and Paigen diet (PD)-fed mice. Single cell suspensions were prepared from solid tissues (aorta, heart, and abdominal fat), and used for total RNA extraction and for qPCR. Relative quantification of IL-17A, IFN-γ, IL-4, TGF-β, IL-1α, IL-12, and IL-6 mRNA levels were performed using the comparative CT method with normalization to GAPDH; results were expressed as fold difference relative to a relevant control sample. **(A)** IL-17 mRNA levels in the aorta, heart and adipose tissue. **(B)** IFN-γ and IL-4 mRNA levels in the aorta. **(C)** IL-1α, IFN-γ, and IL-4 mRNA levels in the heart. **(D)** IL-6 and IL-12 mRNA levels in the adipose tissue. *N* = 3–4. ^*^p < 0.05; ^**^p < 0.01.

Cells of the innate immune system, in particular macrophages, mediate chronic inflammation (Sell et al., 2012). Moreover, B and T lymphocytes of the adaptive immune system have been recently recognized as important modulators of glucose homeostasis, indicating that antigen-driven immune responses could influence insulin resistance. Like macrophages, lymphocytes can be divided into populations with primarily proinflammatory functions (including CD8+ cytotoxic T cells, Th1, Th17) or primarily regulatory functions (including Treg or Th2) and the skewing of the adaptive immune milieu toward a proinflammatory phenotype can exacerbate the metabolic disturbances associated to obesity (Nishimura et al., 2009; Winer et al., 2009; Shen et al., 2015). Moreover, it is known since 1980 that T lymphocyte subsets and related cytokines are present in atherosclerotic lesions and affect their development (Lichtman, 2013). Here, to analyze the changes of adaptive immunity between MSL and MHO, we analyzed T cell populations from the blood and the spleen (secondary lymphoid organ), from the heart and the aorta (cardiovascular tissue), from the liver and from the adipose tissue (metabolic and nutrient hubs) of ND, PD, and HD-fed mice, using the flow cytometry gating strategy depicted in Figure 2. Briefly, CD4+ and CD8+ T cells were identified from the CD45+ lymphocyte populations, whereas to study the myeloid lineage, CD11b+ subpopulation was further analyzed for Ly6G+ cells (neutrophils), CD11b+CD11c+ cells (conventional dendritic cells), and CD11b+CD11c-F4/80+ cells (macrophages; Figure 3). In parallel, we measured by qPCR the intra-tissue expression levels of the following panel of cytokines that play a major role in the adaptive immune system being secreted by helper CD4+ T cells (Th1, Th2, Th17, and Treg) and stimulating several cell types: IL-1α, IL-4, IL-6, IL-12, IL-17 and IFN-γ. IL-1α, IL-12, IL-17, and IFN-γ are generally regarded as pro-inflammatory and pro-atherogenic, while IL-4 and IL-6 display pro- and anti-inflammatory properties which are context-dependent (Hunter and Jones, 2015; Zarzycka et al., 2015). Our analyses showed a great enrichment in myeloid cells, CD45+, CD11b+, CD11c, F480+, Ly6G+ upon PD- in the spleen compared to ND- and HD-feeding (Figure 4A), a massive lymphocyte infiltration in the aorta and in the liver upon both PD and HD compared to ND diet (Figure 4B), a significant increase in CD4+ and CD8+ positive cells percentage exclusively in the aorta and in the hearts of PD-fed animals compared to ND and HD (Figure 4C); no changes were observed in abdominal fat tissues (*data not shown*). At the cytokine level, IL-17 was greatly increased in the aorta, heart and fat only in PD-fed mice compared to HD and ND (Figure 5A). IL-1α, IFN-γ, and IL-4 levels were augmented in the aorta and/or in the heart only in PD-fed mice compared to HD and ND (Figures 5B,C). Finally, we report a trend in increased IL-6 and IL-12 mRNA levels in adipose tissue of PD mice compared to HD- and ND-fed mice (Figure 5D). Collectively, our data surprisingly indicate activation of several components of the adaptive immune system in the metabolic syndrome lean PD mouse model compared to an established mouse model of diet-induced obesity.

### Gut microbiota profiling by metagenomic sequencing

**Figure 6:**
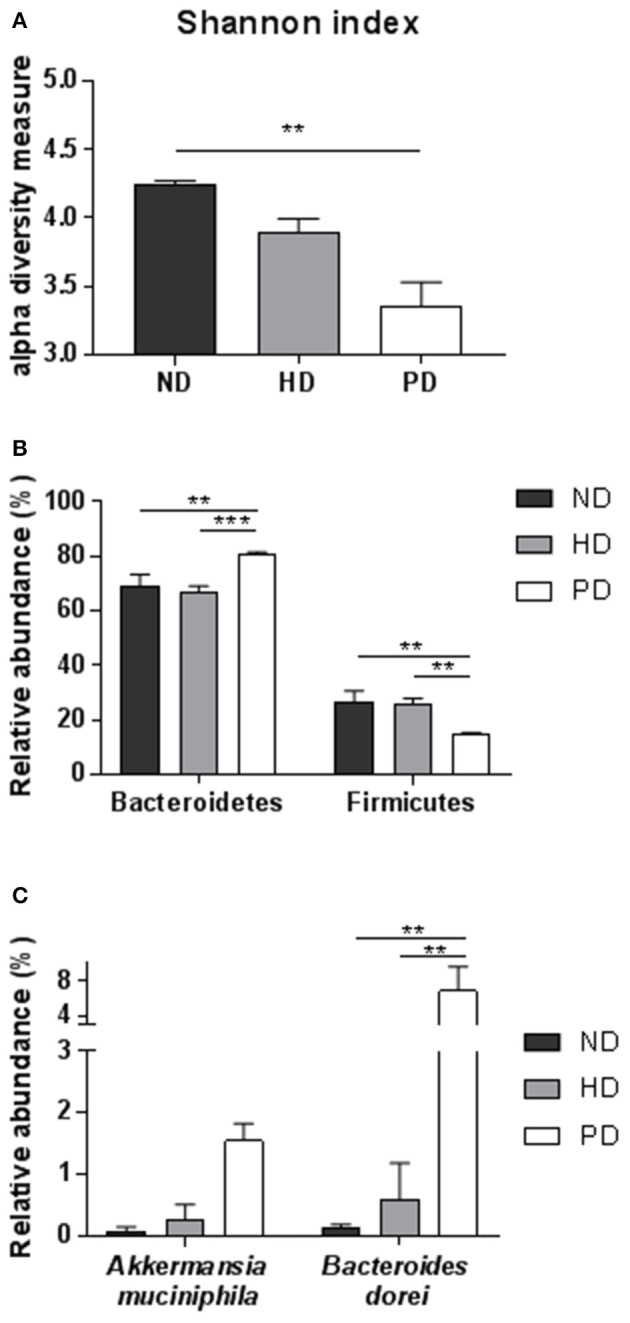
**(A)** Alpha diversity using Shannon index of the fecal microbiota for each groups. **(B)** Relative abundance of major Phylum (Bacteroidetes and Firmicutes) for each group. **(C)** Relative abundance of most significant species, using RDP v11.4 databank in fecal samples of ND, HD, or PD mice. Graphs are displayed as mean ± SEM. ^**^p < 0.01; ^***^p < 0.001, One-Way Anova followed by Kruskal–Wallis test.

**Figure 7:**
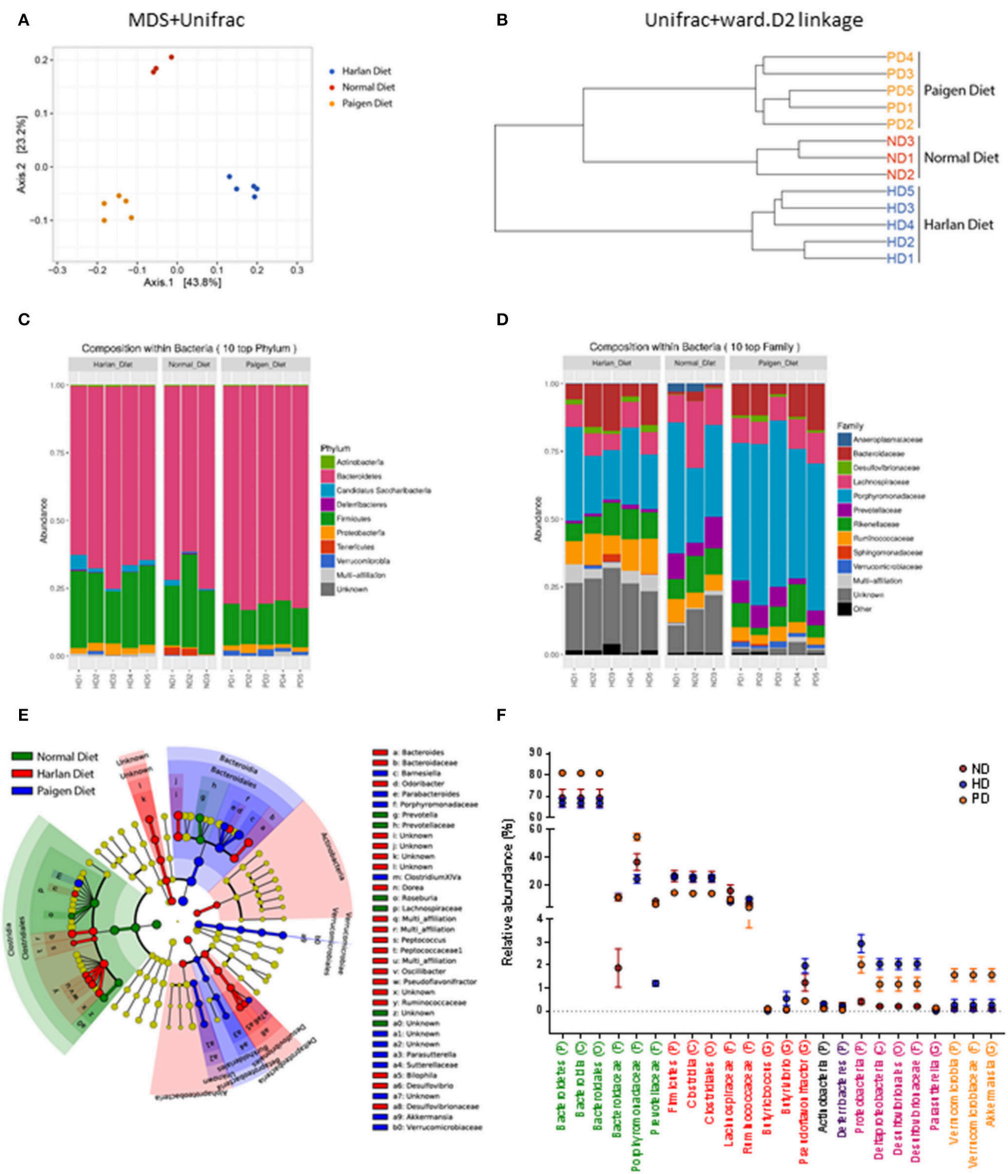
Gut microbiota profiling in Normal diet (ND)-, High fat diet (HD)-, and Paigen diet (PD)-fed mice. **(A)** Multi Dimentional Scaling (MSD) of Unifrac distances of the fecal microbiota for each groups. **(B)** Hierarchical clustering of Unifrac distances of the fecal microbiota for each groups. **(C,D)** Relative abundance of Phylum and Family, respectively for each fecal sample. **(E)** Cladogram representing taxa enriched in fecal samples of ND, HD, or PD mice detected by the LEfSe tool. **(F)** Relative abundance of most significant taxa in fecal samples of ND, HD, or PD mice. Graphs are displayed as mean ± SEM.

The reciprocal interaction between the gut microbiota and the adaptive immunity contributes to the insurgence of metabolic diseases and of inflammation (Kato et al., 2014; Zhang and Luo, 2015; Marchesi et al., 2016). It is however unknown how this interplay adapts to the MSL or MHO clinical features. To this aim we identified bacterial populations contained in fecal samples from ND-, HD-, and PD-fed mice using next generation high throughput sequencing of variable regions (V3–V4) of the 16S rDNA bacterial gene (Lluch et al., 2015; Paisse et al., 2016). Alpha diversity analyses, representing the mean of species diversity in each sample showed that ND-fed mice had a higher taxonomic diversity than the HD-fed mice, which in turn have a higher taxonomic diversity than the PD-fed mice (Figure 6A). Feces microbial composition after 20 weeks of different diet is highly different between the three groups, as shown by beta diversity metrics based multi-dimensional scaling Unifrac analysis (Figure 7A) and by hierarchical clustering (Figure 7B). The community structures observed in the different groups were significantly different. At the phylum level, Firmicutes and Bacteroidetes dominated the fecal microbiota in all groups (Figure 7C). No differences of Firmicutes and Bacteroidetes relative abundance were observed between ND and HD groups (Figure 7C). However, an increase in Bacteroidetes and a decrease in Firmicutes were observed in PD groups (Figure 6B). At the family level, the fecal microbiota was dominated by Porphyromonadaceae in all groups (Figure 7D) and are significantly higher in Paigen Diet mice compared to HD and ND groups (Figure 7F). Focusing on diet effect between the three groups of mice, broad population changes were seen from phylum to genus level (Figure 7E), significantly enriched taxa for all groups are identified using LDA Effect Size (LEfSe) analysis. Clostridia class are significantly enriched in ND and HD mice compared to PD mice (Figures 7E,F). Actinobacteria and Deltaproteobacteria are enriched in HD group compared to ND and PD groups (Figures 7E,F). Bacteroidia and Verrucomicrobia are enriched in PD group compared to ND and HD groups (Figures 7E,F). Interestingly we have identified (with databank RDP v11.4) an increase in *Akkermansia muciniphila* and *Bacteroides dorei* in PD groups compared to HD and ND groups (Figure 6C). Therefore, the most striking result of our metagenomic analyses in gut microbiota composition between the MSL and MHO mimicking diets (PD and HD, respectively) is the preponderance of Bacteroidia and Verrucomicrobia in PD compared to HD and control ND.

### Association of gut microbiota profile with adaptive immune factors

We then sought to explore correlation between changes in gut microbiota composition with the over-responses of the adaptive immune system in mice, irrespective of the diet administered, using linear regression. The associations from regression analyses between gut microbiota classes and cytokines or immune cell types are shown in full in Tables 1–5. Some statistically significant associations were observed in each of the organs analyzed (aorta, heart, adipose tissue, liver, and spleen). For example (1) a linear increase in *Bacteroidia* and a decrease in Clostridiae were associated to an increase in IL-17, IFN-γ, IL-4, and CD8+ cells in the aorta. A decrease in Mollicutes and an increase in Verrucomicrobia was associated to increased infiltration of leukocytes (CD45+) as well as CD4+ and in CD8+ T cells and to an increase in IL-17, in the aorta (Table 1); (2) A linear increase in Bacteroidia and a decrease in Clostridiae was associated to an increase in IFN-γ, IL-6, lymphocytes, and CD4+ cells in the heart. An increase in Verrucomicrobia was associated to increased CD45+ cells and lymphocytes and to an increase in IL-1a, IFN-γ, and in CD4+ cells in the heart (Table 2). (3) A linear decrease in Actinobacteria and in Betaproteobacteria was associate to an increase in IL-6 and/or in IL-12 in the adipose tissue (Table 3). An increase in Verrucomicrobia was associated to increased IL-17 in the adipose tissue (Table 3). (4) A linear increase in Bacteroidia and a decrease in Clostridiae were associated to increased myeloid cells and CD11b+ cells in the liver. Also a decrease in Mollicutes and an increase in Verrucomicrobia was associated to increased myeloid cells in the liver (Table 4). (5) A linear increase in Bacteroidia and in Verrucomicrobia, and a decrease in Clostridiae, were associated to increased myeloid cell markers CD11b+, CD11c+, F4/90+, and Ly6G+ cells in the spleen (Table 5).

**Table 1:** Associations from linear models between gut microbiota and alterations of adaptive immune system parameters measured in the Aorta.

	**IL-17** **β (95% CI)**	**IFNg** **β (95% CI)**	**IL-4** **β (95% CI)**	**TGFb** **β (95% CI)**	**IL-6** **β (95% CI)**	**Lymphocytes** **β (95% CI)**	**CD45+** **β (95% CI)**	**CD4+** **β (95% CI)**	**CD8+** **β (95% CI)**
Actinobacteria	−2.0	−1.7	−1.0	−32.5	−8.9	35.7	25.2	−30.2	−16.5
	(−6.1, 2.1)	(−7.0, 3.5)	(−3.1, 1.0)	(−101.3, 36.3)	(−24.2, 6.5)	(−84.2, 155.5)	(−85.6, 136.1)	(−63.5, 3.1)	(−42.8, 9.8)
	*p* = 0.287	*p* = 0.459	*p* = 0.271	*p* = 0.301	*p* = 0.214	*p* = 0.526	*p* = 0.626	*p* = 0.071	*p* = 0.195
	*R2* = 0.159	*R*^2^ = 0.081	*R*^2^ = 0.169	*R*^2^ = 0.151	*R*^2^ = 0.211	*R*^2^ = 0.037	*R*^2^ = 0.022	*R*^2^ = 0.290	*R*^2^ = 0.147
Alphaproteobacteria	0.1	0.1	0.1	−6.0	0.4	7.8	6.9	0.6	0.8
	(−0.6, 0.8)	(−0.7, 1.0)	(−0.3, 0.4)	(−16.5, 4.5)	(−2.3, 3.1)	(−11.8, 27.5)	(−11.2, 25.0)	(−5.1, 6.2)	(−3.9, 5.5)
	*p* = 0.815	*p* = 0.727	*p* = 0.624	*p* = 0.219	*p* = 0.725	*p* = 0.399	*p* = 0.417	*p* = 0.833	*p* = 0.715
	*R*^2^ = 0.008	*R*^2^ = 0.018	*R*^2^ = 0.036	*R*^2^ = 0.207	*R*^2^ = 0.019	*R*^2^ = 0.066	*R*^2^ = 0.061	*R*^2^ = 0.005	*R*^2^ = 0.013
Bacilli	−0.1	−0.1	−0.0	−1.3	−0.2	−2.1	−2.0	−1.8	−1.6
	(−0.7, 0.5)	(−0.8, 0.6)	(−0.3, 0.3)	(−11.5, 8.8)	(−2.6, 2.1)	(−19.5, 15.3)	(−18.0, 14.0)	(−6.6, 2.9)	(−5.5, 2.4)
	*p* = 0.642	*p* = 0.753	*p* = 0.876	*p* = 0.764	*p* = 0.837	*p* = 0.795	*p* = 0.791	*p* = 0.412	*p* = 0.405
	*R*^2^ = 0.033	*R*^2^ = 0.015	*R*^2^ = 0.004	*R*^2^ = 0.014	*R*^2^ = 0.006	*R*^2^ = 0.006	*R*^2^ = 0.007	*R*^2^ = 0.068	*R*^2^ = 0.064
Bacteroidia	**0.07**	**0.09**	**0.03**	−0.5	**0.2**	0.5	0.3	0.5	**0.4**
	**(0.02, 0.12)**	**(0.02, 0.15)**	**(0.00, 0.06)**	(−1.8, 0.8)	**(0.0, 0.5)**	(−1.4, 2.5)	(−1.5, 2.1)	(−0.0, 0.9)	**(0.0, 0.8)**
	***p* = 0.012**	***p* = 0.018**	***p* = 0.035**	*p* = 0.370	***p* = 0.043**	*p* = 0.564	*p* = 0.684	*p* = 0.055	***p* = 0.043**
	***R***^**2**^ **= 0.615**	***R***^**2**^ **= 0.576**	***R***^**2**^ **= 0.492**	*R*^2^ = 0.116	***R***^**2**^ **= 0.465**	*R*^2^ = 0.031	*R*^2^ = 0.016	*R*^2^ = 0.321	***R***^**2**^ **= 0.323**
Betaproteobacteria	**7.6**	8.3	3.2	9.4	24.4	41.4	30.5	38.5	35.1
	**(0.7, 14.6)**	(−1.1, 17.6)	(−0.8, 7.2)	(−154.1, 172.9)	(−6.4, 55.2)	(−139.0, 221.8)	(−135.7, 196.8)	(−5.2, 82.3)	(−0.6, 70.7)
	***p* = 0.036**	*p* = 0.076	*p* = 0.103	*p* = 0.896	*p* = 0.104	*p* = 0.623	*p* = 0.694	*p* = 0.078	*p* = 0.053
	***R***^**2**^ **= 0.490**	*R*^2^ = 0.382	*R*^2^ = 0.334	*R*^2^ = 0.003	*R*^2^ = 0.333	*R*^2^ = 0.023	*R*^2^ = 0.015	*R*^2^ = 0.278	*R*^2^ = 0.299
Clostridia	**−0.07**	**−0.09**	**−0.03**	1.0	−0.3	−1.0	−0.8	−0.5	**−0.4**
	**(−0.13, −0.01)**	**(−0.16, −0.02)**	**(−0.10, −0.00)**	(−0.2, 2.2)	(−0.5, 0.0)	(−3.1, 1.1)	(−2.7, 1.2)	(−1.0, 0.1)	**(−0.9, −0.0)**
	***p* = 0.021**	***p* = 0.020**	***p* = 0.033**	*p* = 0.094	*p* = 0.052	*p* = 0.305	*p* = 0.409	*p* = 0.073	***p* = 0.044**
	***R***^**2**^ **= 0.554**	***R***^**2**^ **= 0.563**	***R***^**2**^ **= 0.500**	*R*^2^ = 0.349	*R*^2^ = 0.440	*R*^2^ = 0.095	*R*^2^ = 0.063	*R*^2^ = 0.286	***R***^**2**^ **= 0.319**
Deferribacteres	−1.7	−1.8	−0.6	23.0	−3.9	−45.0	−35.1	−18.8	−19.2
	(−5.0, 1.7)	(−6.0, 2.4)	(−2.3, 1.2)	(−35.6, 81.6)	(−17.8, 10.1)	(−142.6, 52.5)	(−125.7, 55.6)	(−45.1, 7.5)	(−39.3, 1.0)
	*p* = 0.285	*p* = 0.349	*p* = 0.483	*p* = 0.384	*p* = 0.533	*p* = 0.332	*p* = 0.413	*p* = 0.142	*p* = 0.060
	*R*^2^ = 0.161	*R*^2^ = 0.126	*R*^2^ = 0.073	*R*^2^ = 0.110	*R*^2^ = 0.058	*R*^2^ = 0.086	*R*^2^ = 0.062	*R*^2^ = 0.202	*R*^2^ = 0.285
Deltaproteobacteria	0.2	0.4	0.2	**−9.0**	1.0	12.0	9.2	−1.3	−0.7
	(−0.5, 0.9)	(−0.4, 1.1)	(−0.2, 0.5)	**(−17.3, −0.7)**	(−1.5, 3.5)	(−3.6, 27.6)	(−5.6, 24.1)	(−6.7, 4.0)	(−4.7, 3.4)
	*p* = 0.496	*p* = 0.298	*p* = 0.267	***p* = 0.037**	*p* = 0.364	*p* = 0.120	*p* = 0.198	*p* = 0.594	*p* = 0.722
	*R*^2^ = 0.069	*R*^2^ = 0.153	*R*^2^ = 0.172	***R***^**2**^ **= 0.485**	*R*^2^ = 0.118	*R*^2^ = 0.205	*R*^2^ = 0.146	*R*^2^ = 0.029	*R*^2^ = 0.012
Erysipelotrichia	−1.0	−1.0	−0.3	−16.5	−3.2	32.6	31.2	−6.6	−4.0
	(−3.4, 1.5)	(−4.1, 2.0)	(−1.6, 0.9)	(−57.5, 24.5)	(−12.8, 6.5)	(−39.8, 105.1)	(−35.0, 97.4)	(−28.2, 14.9)	(−21.5, 13.4)
	*p* = 0.377	*p* = 0.460	*p* = 0.557	*p* = 0.373	*p* = 0.465	*p* = 0.342	*p* = 0.322	*p* = 0.507	*p* = 0.621
	*R*^2^ = 0.113	*R*^2^ = 0.080	*R*^2^ = 0.051	*R*^2^ = 0.114	*R*^2^ = 0.079	*R*^2^ = 0.082	*R*^2^ = 0.089	*R*^2^ = 0.045	*R*^2^ = 0.023
Gammaproteobacteria	3.5	4.9	−1.9	−118.3	−21.9	106.9	71.4	−28.1	−4.8
	(−11.1, 18.0)	(−12.8, 22.6)	(−9.2, 5.4)	(−345.4, 108.7)	(−76.3, 32.4)	(−106.6, 320.4)	(−129.6, 272.3)	(−88.3, 32.1)	(−57.2, 47.6)
	*p* = 0.590	*p* = 0.534	*p* = 0.558	*p* = 0.257	*p* = 0.372	*p* = 0.294	*p* = 0.451	*p* = 0.324	*p* = 0.845
	*R*^2^ = 0.044	*R*^2^ = 0.058	*R*^2^ = 0.051	*R*^2^ = 0.178	*R*^2^ = 0.115	*R*^2^ = 0.100	*R*^2^ = 0.053	*R*^2^ = 0.097	*R*^2^ = 0.004
Mollicutes	−0.2	−0.3	−0.1	5.6	−0.7	**−14.9**	**−12.5**	−1.7	−2.2
	(−0.7, 0.2)	(−0.9, 0.3)	(−0.4, 0.1)	(−1.5, 12.7)	(−2.6, 1.2)	**(−25.9, −3.9)**	**(−23.3, −1.7)**	(−5.7, 2.3)	(−5.3, 0.9)
	*p* = 0.298	*p* = 0.269	*p* = 0.276	*p* = 0.105	*p* = 0.414	***p* = 0.013**	***p* = 0.027**	*p* = 0.370	*p* = 0.150
	*R*^2^ = 0.153	*R*^2^ = 0.171	*R*^2^ = 0.167	*R*^2^ = 0.331	*R*^2^ = 0.097	***R***^**2**^ **= 0.445**	***R***^**2**^ **= 0.370**	*R*^2^ = 0.081	*R*^2^ = 0.179
Multi-affiliation	−1.4	−1.5	−0.6	−9.2	−4.8	10.2	7.2	−0.6	0.9
	(−3.5, 0.7)	(−4.2, 1.2)	(−1.7, 0.6)	(−49.7, 31.3)	(−13.3, 3.7)	(−20.9, 41.3)	(−21.6, 36.0)	(−9.4, 8.3)	(−6.5, 8.3)
	*p* = 0.161	*p* = 0.236	*p* = 0.270	*p* = 0.608	*p* = 0.220	*p* = 0.484	*p* = 0.595	*p* = 0.891	*p* = 0.799
	*R*^2^ = 0.260	*R*^2^ = 0.193	*R*^2^ = 0.170	*R*^2^ = 0.040	*R*^2^ = 0.206	*R*^2^ = 0.045	*R*^2^ = 0.026	*R*^2^ = 0.002	*R*^2^ = 0.006
Negativicutes	2.1	2.7	−0.3	−32.4	−4.2	52.8	35.8	2.3	8.4
	(−2.6, 6.7)	(−3.0, 8.3)	(−2.8, 2.2)	(−111.5, 46.8)	(−23.2, 14.9)	(−87.0, 192.6)	(−94.3, 165.9)	(−45.7, 50.3)	(−24.7, 41.6)
	*p* = 0.332	*p* = 0.297	*p* = 0.792	*p* = 0.366	*p* = 0.622	*p* = 0.423	*p* = 0.557	*p* = 0.918	*p* = 0.587
	*R*^2^ = 0.134	*R*^2^ = 0.154	*R*^2^ = 0.011	*R*^2^ = 0.118	*R*^2^ = 0.037	*R*^2^ = 0.059	*R*^2^ = 0.032	*R*^2^ = 0.001	*R*^2^ = 0.028
Verrucomicrobiae	**0.5**	0.6	0.2	−6.4	1.5	14.9	13.8	**6.4**	**5.5**
	**(0.1, 1.0)**	(−0.0, 1.2)	(−0.1, 0.5)	(−16.2, 3.3)	(−0.7, 3.8)	(−0.3, 30.2)	(−0.2, 27.8)	**(3.9, 8.9)**	**(3.4, 7.7)**
	***p* = 0.027**	*p* = 0.057	*p* = 0.100	*p* = 0.164	*p* = 0.147	*p* = 0.054	*p* = 0.052	**p< 0.001**	**p< 0.001**
	***R***^**2**^ **= 0.526**	*R*^2^ = 0.425	*R*^2^ = 0.338	*R*^2^ = 0.257	*R*^2^ = 0.276	*R*^2^ = 0.297	*R*^2^ = 0.301	***R***^**2**^ **= 0.761**	***R***^**2**^ **= 0.750**

*In each of the models, the associations are reported as absolute difference (β), with 95% Confidence Intervals (CI), in immune system parameters levels by 1 unit increase in gut microbiota. In bold are reported the statistically significant results (p < 0.05). Per each of the models, the Coefficient of determination (R^2^) is also reported*.

**Table 2:** Associations from linear regression models between gut microbiota and alterations of adaptive immune system parameters measured in the heart.

	**IL-17** **β (95% CI)**	**IFNg** **β (95% CI)**	**IL-4** **β (95% CI)**	**IL-1α** **β (95% CI)**	**IL-6** **β (95% CI)**	**Lymphocytes** **β (95% CI)**	**CD45+** **β (95% CI)**	**CD4+** **β (95% CI)**	**CD8+** **β (95% CI)**
Actinobacteria	−1.6	−1.8	−3	−1.5	−6	65	55.1	**−30.9**	−7.1
	(−8.4, 5.2)	(−4.7, 1.1)	(−8.6, 2.5)	(−4.9, 1.9)	(−15.0, 3.0)	(−39.7, 169.7)	(−45.7, 156.0)	**(−58.7, −3.1)**	(−35.7, 21.5)
	*p* = 0.602	*p* = 0.178	*p* = 0.201	*p* = 0.321	*p* = 0.157	*p* = 0.199	*p* = 0.254	***p* = 0.032**	*p* = 0.597
	*R*^2^ = 0.041	*R*^2^ = 0.243	*R*^2^ = 0.368	*R*^2^ = 0.140	*R*^2^ = 0.264	*R*^2^ = 0.145	*R*^2^ = 0.116	***R***^**2**^ **= 0.353**	*R*^2^ = 0.026
Alphaproteobacteria	0.1	0	−0.1	0	0.2	−1.1	3.4	0.4	1.1
	(−1.0, 1.2)	(−0.5, 0.6)	(−1.1, 0.9)	(−0.6, 0.5)	(−1.4, 1.9)	(−20.0, 17.7)	(−14.3, 21.1)	(−5.3, 6.2)	(−3.7, 5.9)
	*p* = 0.882	*p* = 0.900	*p* = 0.751	*p* = 0.848	*p* = 0.773	*p* = 0.896	*p* = 0.680	*p* = 0.879	*p* = 0.625
	*R*^2^ = 0.003	*R*^2^ = 0.002	*R*^2^ = 0.028	*R*^2^ = 0.006	*R*^2^ = 0.013	*R*^2^ = 0.002	*R*^2^ = 0.016	*R*^2^ = 0.002	*R*^2^ = 0.022
Bacilli	−0.1	−0.1	−0.2	−0.2	−0.2	2.9	1.9	−1.2	−0.3
	(−1.1, 0.8)	(−0.6, 0.3)	(−1.0, 0.6)	(−0.7, 0.2)	(−1.7, 1.2)	(−13.2, 19.0)	(−13.5, 17.2)	(−6.1, 3.7)	(−4.4, 3.9)
	*p* = 0.740	*p* = 0.570	*p* = 0.485	*p* = 0.283	*p* = 0.709	*p* = 0.697	*p* = 0.795	*p* = 0.597	*p* = 0.894
	*R*^2^ = 0.017	*R*^2^ = 0.048	*R*^2^ = 0.129	*R*^2^ = 0.162	*R*^2^ = 0.021	*R*^2^ = 0.014	*R*^2^ = 0.006	*R*^2^ = 0.026	*R*^2^ = 0.002
Bacteroidia	0.1	**0.1**	**0.2**	0	**0.2**	**−1.6**	−0.6	**0.6**	0.3
	(−0.0, 0.2)	**(0.0, 0.1)**	**(0.1, 0.3)**	(−0.0, 0.1)	**(0.0, 0.3)**	**(−3.1, −0.2)**	(−2.3, 1.1)	**(0.2, 1.0)**	(−0.1, 0.7)
	*p* = 0.094	***p* = 0.011**	***p* = 0.014**	*p* = 0.053	***p* = 0.033**	***p* = 0.034**	*p* = 0.490	***p* = 0.009**	*p* = 0.119
	*R*^2^ = 0.349	***R***^**2**^ **= 0.628**	***R***^**2**^ **= 0.814**	*R*^2^ = 0.437	***R***^**2**^ **= 0.501**	***R***^**2**^ **= 0.349**	*R*^2^ = 0.044	***R***^**2**^ **= 0.480**	*R*^2^ = 0.207
Betaproteobacteria	8.1	**5.9**	8.1	**6.1**	16.7	−98.3	−28.3	**58.5**	12.5
	(−5.3, 21.5)	**(0.8, 10.9)**	(−2.0, 18.1)	**(0.4, 11.9)**	(−0.7, 34.2)	(−254.5, 57.8)	(−187.4, 130.9)	**(24.4, 92.5)**	(−30.0, 55.0)
	*p* = 0.196	***p* = 0.029**	*p* = 0.090	***p* = 0.041**	*p* = 0.058	*p* = 0.193	*p* = 0.703	***p* = 0.003**	*p* = 0.531
	*R*^2^ = 0.226	***R***^**2**^ **= 0.518**	*R*^2^ = 0.553	***R***^**2**^ **= 0.473**	*R*^2^ = 0.423	*R*^2^ = 0.149	*R*^2^ = 0.014	***R***^**2**^ **= 0.564**	*R*^2^ = 0.037
Clostridia	−0.1	**−0.1**	−0.1	0	**−0.2**	**1.7**	0.5	**−0.6**	−0.4
	(−0.2, 0.0)	**(−0.1, −0.0)**	(−0.3, 0.0)	(−0.1, 0.0)	**(−0.3, −0.0)**	**(0.1, 3.4)**	(−1.4, 2.4)	**(−1.1, −0.1)**	(−0.8, 0.1)
	*p* = 0.095	***p* = 0.023**	*p* = 0.086	*p* = 0.083	***p* = 0.045**	***p* = 0.042**	*p* = 0.544	***p* = 0.017**	*p* = 0.113
	*R*^2^ = 0.347	***R***^**2**^ **= 0.547**	*R*^2^ = 0.563	*R*^2^ = 0.369	***R***^**2**^ **= 0.458**	***R***^**2**^ **= 0.324**	*R*^2^ = 0.034	***R***^**2**^ **= 0.417**	*R*^2^ = 0.212
Deferribacteres	−0.7	−1.1	4.5	−1.2	−3	34.3	−23.7	−15.9	−9.8
	(−6.4, 5.1)	(−3.7, 1.4)	(−9.2, 18.2)	(−4.1, 1.6)	(−11.3, 5.3)	(−57.5, 126.1)	(−111.9, 64.5)	(−42.8, 10.9)	(−33.1, 13.5)
	*p* = 0.789	*p* = 0.323	*p* = 0.411	*p* = 0.334	*p* = 0.424	*p* = 0.428	*p* = 0.566	*p* = 0.218	*p* = 0.375
	*R*^2^ = 0.011	*R*^2^ = 0.139	*R*^2^ = 0.174	*R*^2^ = 0.133	*R*^2^ = 0.093	*R*^2^ = 0.058	*R*^2^ = 0.031	*R*^2^ = 0.134	*R*^2^ = 0.072
Deltaproteobacteria	0.7	0.2	0.7	0.2	0.5	−2.2	−6.5	−0.5	0.3
	(−0.1, 1.6)	(−0.3, 0.6)	(−0.2, 1.5)	(−0.4, 0.7)	(−1.1, 2.0)	(−18.4, 13.9)	(−21.2, 8.3)	(−5.4, 4.4)	(−3.8, 4.5)
	*p* = 0.074	*p* = 0.485	*p* = 0.086	*p* = 0.516	*p* = 0.490	*p* = 0.767	*p* = 0.356	*p* = 0.829	*p* = 0.870
	*R*^2^ = 0.387	*R*^2^ = 0.072	*R*^2^ = 0.562	*R*^2^ = 0.063	*R*^2^ = 0.070	*R*^2^ = 0.008	*R*^2^ = 0.078	*R*^2^ = 0.004	*R*^2^ = 0.003
Erysipelotrichia	−0.8	−0.8	−66.6	−0.7	−2	26.4	3.5	−8.8	−5.9
	(−4.8, 3.2)	(−2.6, 1.1)	(−257.8, 124.7)	(−2.7, 1.3)	(−7.9, 3.8)	(−41.5, 94.2)	(−62.8, 69.8)	(−29.4, 11.7)	(−23.4, 11.7)
	*p* = 0.636	*p* = 0.359	*p* = 0.389	*p* = 0.434	*p* = 0.436	*p* = 0.411	*p* = 0.910	*p* = 0.365	*p* = 0.477
	*R*^2^ = 0.034	*R*^2^ = 0.121	*R*^2^ = 0.189	*R*^2^ = 0.090	*R*^2^ = 0.089	*R*^2^ = 0.062	*R*^2^ = 0.001	*R*^2^ = 0.075	*R*^2^ = 0.047
Gammaproteobacteria	−5	−0.4	−5.3	−2.5	−11.9	4.1	35.4	−35.8	10.9
	(−27.8, 17.9)	(−11.5, 10.7)	(−23.8, 13.2)	(−14.5, 9.5)	(−45.4, 21.7)	(−204.5, 212.7)	(−160.7, 231.6)	(−94.8, 23.3)	(−42.0, 63.8)
	*p* = 0.624	*p* = 0.939	*p* = 0.473	*p* = 0.639	*p* = 0.430	*p* = 0.966	*p* = 0.699	*p* = 0.209	*p* = 0.659
	*R*^2^ = 0.036	*R*^2^ = 0.001	*R*^2^ = 0.136	*R*^2^ = 0.033	*R*^2^ = 0.091	*R*^2^ = 0.000	*R*^2^ = 0.014	*R*^2^ = 0.139	*R*^2^ = 0.018
Mollicutes	−0.3	−0.2	−0.3	−0.2	−0.5	3.9	1	−1.5	−1.1
	(−1.1, 0.4)	(−0.5, 0.2)	(−1.1, 0.5)	(−0.6, 0.2)	(−1.6, 0.7)	(−9.6, 17.4)	(−12.0, 14.0)	(−5.6, 2.6)	(−4.5, 2.4)
	*p* = 0.322	*p* = 0.329	*p* = 0.373	*p* = 0.398	*p* = 0.378	*p* = 0.540	*p* = 0.868	*p* = 0.433	*p* = 0.504
	*R*^2^ = 0.140	*R*^2^ = 0.136	*R*^2^ = 0.201	*R*^2^ = 0.104	*R*^2^ = 0.113	*R*^2^ = 0.035	*R*^2^ = 0.003	*R*^2^ = 0.057	*R*^2^ = 0.042
Multi-affiliation	−1.3	−1.1	−4.9	−1.1	−3.2	6.7	5.6	0.4	−2.2
	(−4.9, 2.4)	(−2.6, 0.5)	(−13.7, 3.9)	(−2.8, 0.7)	(−8.3, 1.8)	(−22.5, 35.8)	(−22.0, 33.3)	(−8.6, 9.4)	(−9.6, 5.2)
	*p* = 0.443	*p* = 0.139	*p* = 0.196	*p* = 0.192	*p* = 0.177	*p* = 0.625	*p* = 0.662	*p* = 0.922	*p* = 0.527
	*R*^2^ = 0.086	*R*^2^ = 0.285	*R*^2^ = 0.375	*R*^2^ = 0.230	*R*^2^ = 0.244	*R*^2^ = 0.022	*R*^2^ = 0.018	*R*^2^ = 0.001	*R*^2^ = 0.037
Negativicutes	1.3	0.8	0.1	1.3	−1.8	−68.7	35.8	−19.6	**32.3**
	(−6.5, 9.0)	(−2.9, 4.5)	(−7.5, 7.8)	(−2.7, 5.2)	(−13.5, 10.0)	(−194.3, 57.0)	(−88.6, 160.1)	(−58.3, 19.0)	**(5.7, 59.0)**
	*p* = 0.706	*p* = 0.629	*p* = 0.964	*p* = 0.470	*p* = 0.732	*p* = 0.254	*p* = 0.540	*p* = 0.287	***p* = 0.022**
	*R*^2^ = 0.022	*R*^2^ = 0.035	*R*^2^ = 0.001	*R*^2^ = 0.077	*R*^2^ = 0.018	*R*^2^ = 0.116	*R*^2^ = 0.035	*R*^2^ = 0.102	***R***^**2**^ **= 0.394**
Verrucomicrobiae	0.6	**0.4**	0.4	**0.6**	1.2	−10.6	2.9	**4.4**	3.1
	(−0.4, 1.5)	**(0.1, 0.8)**	(−0.5, 1.2)	**(0.4, 0.8)**	(−0.1, 2.4)	(−25.9, 4.8)	(−13.0, 18.8)	**(0.1, 8.6)**	(−0.7, 6.9)
	*p* = 0.195	***p* = 0.025**	*p* = 0.303	***p* = 0.000**	*p* = 0.058	*p* = 0.158	*p* = 0.696	***p* = 0.044**	*p* = 0.097
	*R*^2^ = 0.227	***R***^**2**^ **= 0.534**	*R*^2^ = 0.258	***R***^**2**^ **= 0.850**	*R*^2^ = 0.423	*R*^2^ = 0.173	*R*^2^ = 0.014	***R***^**2**^ **= 0.319**	R2 = 0.231

*In each of the models, the associations are reported as absolute difference (β), with 95% Confidence Intervals (CI), in immune system parameters levels by 1 unit increase in gut microbiota. In bold are reported the statistically significant results (p < 0.05). Per each of the models, the Coefficient of determination (R^2^) is also reported*.

**Table 3:** Associations from linear regression models between gut microbiota and alterations of adaptive immune system parameters measured in the adipose tissue.

	**IL-17** **β (95% CI)**	**IL-12** **β (95% CI)**	**IL-6** **β (95% CI)**
Actinobacteria	−1.6	**−1.6**	−1.9
	(−3.4, 0.1)	**(−2.9, −0.4)**	(−4.1, 0.2)
	*p* = 0.065	***p* = 0.020**	*p* = 0.071
	*R*^2^ = 0.407	***R***^**2**^ **= 0.564**	*R*^2^ = 0.393
Alphaproteobacteria	0	0	0
	(−0.4, 0.3)	(−0.3, 0.3)	(−0.5, 0.4)
	*p* = 0.761	*p* = 0.841	*p* = 0.799
	*R*^2^ = 0.014	*R*^2^ = 0.006	*R*^2^ = 0.010
Bacilli	−0.1	−0.1	−0.1
	(−0.4, 0.1)	(−0.3, 0.2)	(−0.5, 0.3)
	*p* = 0.294	*p* = 0.470	*p* = 0.561
	*R*^2^ = 0.155	*R*^2^ = 0.077	*R*^2^ = 0.050
Bacteroidia	0.02	0.02	0.04
	(−0.01, 0.06)	(−0.01, 0.05)	(−0.00, 0.07)
	*p* = 0.186	*p* = 0.093	*p* = 0.072
	*R*^2^ = 0.235	*R*^2^ = 0.350	*R*^2^ = 0.390
Betaproteobacteria	**5.1**	**3.7**	4.4
	**(3.0, 7.2)**	**(1.0, 6.4)**	(−0.2, 9.1)
	***p* = 0.001**	***p* = 0.014**	*p* = 0.058
	***R***^**2**^ **= 0.830**	***R***^**2**^ **= 0.599**	*R*^2^ = 0.423
Clostridia	0	0	0
	(−0.1, 0.0)	(−0.1, 0.0)	(−0.1, 0.0)
	*p* = 0.315	*p* = 0.185	*p* = 0.148
	*R*^2^ = 0.143	*R*^2^ = 0.236	*R*^2^ = 0.274
Deferribacteres	−0.4	−0.3	−0.3
	(−2.3, 1.5)	(−1.9, 1.3)	(−2.6, 2.0)
	*p* = 0.633	*p* = 0.660	*p* = 0.739
	*R*^2^ = 0.034	*R*^2^ = 0.029	*R*^2^ = 0.017
Deltaproteobacteria	−0.1	0	0.1
	(−0.4, 0.3)	(−0.3, 0.3)	(−0.3, 0.5)
	*p* = 0.685	*p* = 0.905	*p* = 0.525
	*R*^2^ = 0.025	*R*^2^ = 0.002	*R*^2^ = 0.060
Erysipelotrichia	−0.5	−0.4	−0.5
	(−1.8, 0.7)	(−1.5, 0.7)	(−2.1, 1.0)
	*p* = 0.367	*p* = 0.412	*p* = 0.452
	*R*^2^ = 0.117	*R*^2^ = 0.098	*R*^2^ = 0.083
Gammaproteobacteria	0.4	−2.6	−2.5
	(−7.3, 8.0)	(−8.7, 3.5)	(−11.5, 6.6)
	*p* = 0.908	*p* = 0.346	*p* = 0.540
	*R*^2^ = 0.002	*R*^2^ = 0.127	*R*^2^ = 0.056
Mollicutes	0	−0.1	−0.1
	(−0.3, 0.2)	(−0.3, 0.2)	(−0.4, 0.2)
	*p* = 0.840	*p* = 0.580	*p* = 0.471
	*R*^2^ = 0.006	*R*^2^ = 0.046	*R*^2^ = 0.077
Multi-affiliation	−0.8	−0.6	−0.7
	(−1.8, 0.2)	(−1.5, 0.3)	(−2.1, 0.7)
	*p* = 0.111	*p* = 0.157	*p* = 0.252
	*R*^2^ = 0.321	*R*^2^ = 0.264	*R*^2^ = 0.182
Negativicutes	0.7	−0.4	−0.1
	(−1.8, 3.2)	(−2.6, 1.7)	(−3.2, 3.1)
	*p* = 0.550	*p* = 0.654	*p* = 0.965
	*R*^2^ = 0.053	*R*^2^ = 0.030	*R*^2^ = 0.000
Verrucomicrobiae	**0.3**	0.2	0.2
	**(0.1, 0.5)**	(−0.1, 0.4)	(−0.2, 0.6)
	***p* = 0.022**	*p* = 0.106	*p* = 0.258
	***R***^**2**^ **= 0.551**	*R*^2^ = 0.330	*R*^2^ = 0.178

*In each of the models, the associations are reported as absolute difference (β), with 95% Confidence Intervals (CI), in immune system parameters levels by 1 unit increase in gut microbiota. In bold are reported the statistically significant results (p < 0.05). Per each of the models, the Coefficient of determination (R^2^) is also reported*.

**Table 4:** Associations from linear regression models between gut microbiota and alterations of adaptive immune system parameters measured in the liver.

	**Myeloid cells** **β (95% CI)**	**Lymphocytes** **β (95% CI)**	**CD4+** **β (95% CI)**	**CD8+** **β (95% CI)**	**CD45+** **β (95% CI)**	**CD11b+** **β (95% CI)**	**CD11c+** **β (95% CI)**	**F480+** **β (95% CI)**	**Ly6G+** **β (95% CI)**
Actinobacteria	−14.9	−6.3	0.5	0.6	0.4	−25.6	10.4	−36.5	−70.7
	(−67.9, 38.1)	(−128.4, 115.7)	(−18.9, 19.9)	(−10.9, 12.1)	(−70.6, 71.4)	(−55.4, 4.1)	(−32.8, 53.7)	(−77.9, 4.9)	(−167.3, 26.0)
	*p* = 0.549	*p* = 0.911	*p* = 0.952	*p* = 0.912	*p* = 0.991	*p* = 0.085	*p* = 0.606	*p* = 0.079	*p* = 0.136
	*R*^2^ = 0.034	*R*^2^ = 0.001	*R*^2^ = 0.000	*R*^2^ = 0.001	*R*^2^ = 0.000	*R*^2^ = 0.246	*R*^2^ = 0.025	*R*^2^ = 0.255	*R*^2^ = 0.191
Alphaproteobacteria	5.7	8.8	0.9	0.7	4.1	1.4	1.8	−1.8	−12.7
	(−2.4, 13.9)	(−10.7, 28.3)	(−2.3, 4.1)	(−1.2, 2.6)	(−7.4, 15.6)	(−4.3, 7.0)	(−5.4, 9.0)	(−9.7, 6.1)	(−28.5, 3.0)
	*p* = 0.148	*p* = 0.341	*p* = 0.539	*p* = 0.421	*p* = 0.444	*p* = 0.601	*p* = 0.587	*p* = 0.627	*p* = 0.103
	*R*^2^ = 0.180	*R*^2^ = 0.083	*R*^2^ = 0.035	*R*^2^ = 0.060	*R*^2^ = 0.054	*R*^2^ = 0.026	*R*^2^ = 0.028	*R*^2^ = 0.022	*R*^2^ = 0.223
Bacilli	2.3	−0.8	−0.2	−0.1	−1.4	0.5	0.4	−1.5	−6.3
	(−5.3, 9.9)	(−18.3, 16.7)	(−3.0, 2.6)	(−1.7, 1.6)	(−11.5, 8.7)	(−4.4, 5.4)	(−5.8, 6.7)	(−8.3, 5.3)	(−21.1, 8.5)
	*p* = 0.516	*p* = 0.922	*p* = 0.863	*p* = 0.901	*p* = 0.765	*p* = 0.814	*p* = 0.884	*p* = 0.644	*p* = 0.367
	*R*^2^ = 0.039	*R*^2^ = 0.001	*R*^2^ = 0.003	*R*^2^ = 0.002	*R*^2^ = 0.009	*R*^2^ = 0.005	*R*^2^ = 0.002	*R*^2^ = 0.020	*R*^2^ = 0.074
Bacteroidia	**1.2**	1.6	0.2	0.1	0.8	**0.6**	0.2	0.5	−0.7
	**(0.7, 1.6)**	(−0.0, 3.3)	(−0.1, 0.5)	(−0.1, 0.3)	(−0.2, 1.8)	**(0.2, 1.0)**	(−0.5, 0.9)	(−0.3, 1.2)	(−2.4, 0.9)
	**P< 0.001**	*p* = 0.054	*p* = 0.172	*p* = 0.264	*p* = 0.115	***p* = 0.004**	*p* = 0.490	*p* = 0.183	*p* = 0.359
	***R***^**2**^ **= 0.773**	*R*^2^ = 0.297	*R*^2^ = 0.163	*R*^2^ = 0.112	*R*^2^ = 0.210	***R***^**2**^ **= 0.551**	*R*^2^ = 0.044	*R*^2^ = 0.155	*R*^2^ = 0.077
Betaproteobacteria	51.6	115.5	14.1	5.6	55.2	38.3	40.9	11.6	−33.2
	(−21.3, 124.5)	(−50.0, 281.0)	(−13.3, 41.6)	(−11.2, 22.5)	(−44.4, 154.7)	(−6.2, 82.7)	(−18.6, 100.4)	(−59.6, 82.8)	(−192.2, 125.7)
	*p* = 0.148	*p* = 0.153	*p* = 0.281	*p* = 0.478	*p* = 0.248	*p* = 0.085	*p* = 0.159	*p* = 0.727	*p* = 0.654
	*R*^2^ = 0.180	*R*^2^ = 0.177	*R*^2^ = 0.105	*R*^2^ = 0.047	*R*^2^ = 0.119	*R*^2^ = 0.246	*R*^2^ = 0.172	*R*^2^ = 0.012	*R*^2^ = 0.019
Clostridia	**−1.3**	**−1.9**	−0.2	−0.1	−0.9	**−0.6**	−0.3	−0.3	1
	**(−1.8, −0.8)**	**(−3.7, −0.1)**	(−0.5, 0.1)	(−0.3, 0.1)	(−2.0, 0.2)	**(−1.1, −0.1)**	(−1.1, 0.4)	(−1.2, 0.5)	(−0.8, 2.8)
	**p< 0.001**	***p* = 0.044**	*p* = 0.169	*p* = 0.234	*p* = 0.099	***p* = 0.019**	*p* = 0.369	*p* = 0.379	*p* = 0.234
	***R***^**2**^ **= 0.755**	***R***^**2**^ **= 0.319**	*R*^2^ = 0.164	*R*^2^ = 0.126	*R*^2^ = 0.228	***R***^**2**^ **= 0.406**	*R*^2^ = 0.074	*R*^2^ = 0.071	*R*^2^ = 0.126
Deferribacteres	**−50.6**	−66.7	−10.1	−6.6	−37.3	**−29.3**	−5.6	−20	44
	**(−80.6, −20.6)**	(−158.6, 25.1)	(−24.9, 4.6)	(−15.2, 2.0)	(−91.2, 16.6)	**(−50.3, −8.4)**	(−42.0, 30.8)	(−57.8, 17.8)	(−40.9, 128.8)
	***p* = 0.003**	*p* = 0.138	*p* = 0.159	*p* = 0.120	*p* = 0.156	***p* = 0.011**	*p* = 0.741	*p* = 0.269	*p* = 0.278
	***R***^**2**^ **= 0.555**	*R*^2^ = 0.189	*R*^2^ = 0.172	*R*^2^ = 0.205	*R*^2^ = 0.174	***R***^**2**^ **= 0.463**	*R*^2^ = 0.010	*R*^2^ = 0.110	*R*^2^ = 0.106
Deltaproteobacteria	0.9	10.6	1.7	0.9	6.3	−2.8	1.2	−3.8	−10.1
	(−6.9, 8.6)	(−5.4, 26.6)	(−0.8, 4.3)	(−0.6, 2.5)	(−3.0, 15.6)	(−7.4, 1.7)	(−5.0, 7.5)	(−10.2, 2.6)	(−24.0, 3.8)
	*p* = 0.812	*p* = 0.173	*p* = 0.165	*p* = 0.201	*p* = 0.165	*p* = 0.201	*p* = 0.672	*p* = 0.219	*p* = 0.137
	*R*^2^ = 0.005	*R*^2^ = 0.162	*R*^2^ = 0.168	*R*^2^ = 0.144	*R*^2^ = 0.167	*R*^2^ = 0.144	*R*^2^ = 0.017	*R*^2^ = 0.134	*R*^2^ = 0.190
Erysipelotrichia	−23.4	−48.7	−7.2	−3.6	−26.3	−11.8	−0.1	−11.1	28.4
	(−52.9, 6.1)	(−117.0, 19.6)	(−18.2, 3.9)	(−10.3, 3.2)	(−66.6, 14.0)	(−31.5, 7.9)	(−27.2, 27.0)	(−39.9, 17.6)	(−35.3, 92.1)
	*p* = 0.109	*p* = 0.145	*p* = 0.181	*p* = 0.267	*p* = 0.179	*p* = 0.215	*p* = 0.996	*p* = 0.412	*p* = 0.348
	*R*^2^ = 0.216	*R*^2^ = 0.183	*R*^2^ = 0.157	*R*^2^ = 0.111	*R*^2^ = 0.158	*R*^2^ = 0.136	*R*^2^ = 0.000	*R*^2^ = 0.062	*R*^2^ = 0.080
Gammaproteobacteria	13.6	123.7	**30.8**	16.7	86.8	−11.7	−0.3	−27.4	−100.9
	(−85.3, 112.6)	(−85.7, 333.0)	**(1.5, 60.1)**	(−1.5, 34.8)	(−30.6, 204.2)	(−74.4, 50.9)	(−81.0, 80.3)	(−113.8, 59.1)	(−287.0, 85.3)
	*p* = 0.767	*p* = 0.220	***p* = 0.041**	*p* = 0.068	*p* = 0.132	*p* = 0.688	*p* = 0.993	*p* = 0.501	*p* = 0.258
	*R*^2^ = 0.008	*R*^2^ = 0.133	***R***^**2**^ **= 0.327**	*R*^2^ = 0.271	*R*^2^ = 0.194	*R*^2^ = 0.015	*R*^2^ = 0.000	*R*^2^ = 0.042	*R*^2^ = 0.115
Mollicutes	**−6**	−10.7	−1.7	**−1.2**	−6.2	−2.9	−0.5	−1.4	8
	**(−11.2, −0.8)**	(−23.7, 2.3)	(−3.8, 0.4)	**(−2.3, −0.0)**	(−13.8, 1.4)	(−6.6, 0.7)	(−5.8, 4.8)	(−7.1, 4.4)	(−3.9, 19.9)
	***p* = 0.028**	*p* = 0.098	*p* = 0.098	***p* = 0.049**	*p* = 0.099	*p* = 0.107	*p* = 0.838	*p* = 0.610	*p* = 0.169
	***R***^**2**^ **= 0.370**	*R*^2^ = 0.229	*R*^2^ = 0.229	***R***^**2**^ **= 0.308**	*R*^2^ = 0.228	*R*^2^ = 0.219	*R*^2^ = 0.004	*R*^2^ = 0.024	*R*^2^ = 0.165
Multi−affiliation	−2.6	9.6	2.1	1.3	8.3	0.7	−1.4	−1.2	−4
	(−16.5, 11.4)	(−21.6, 40.7)	(−2.7, 7.0)	(−1.6, 4.2)	(−9.3, 26.0)	(−8.2, 9.6)	(−12.8, 9.9)	(−13.7, 11.2)	(−31.8, 23.9)
	*p* = 0.693	*p* = 0.513	*p* = 0.355	*p* = 0.333	*p* = 0.321	*p* = 0.869	*p* = 0.788	*p* = 0.831	*p* = 0.758
	*R*^2^ = 0.015	*R*^2^ = 0.040	*R*^2^ = 0.078	*R*^2^ = 0.085	*R*^2^ = 0.089	*R*^2^ = 0.003	*R*^2^ = 0.007	*R*^2^ = 0.004	*R*^2^ = 0.009
Negativicutes	23.9	35.5	6.8	−2.2	25.8	10.6	−11.8	31.1	8.6
	(−37.8, 85.5)	(−106.6, 177.6)	(−15.7, 29.3)	(−15.8, 11.3)	(−56.2, 107.8)	(−29.2, 50.4)	(−62.9, 39.2)	(−21.6, 83.8)	(−118.0, 135.2)
	*p* = 0.412	*p* = 0.593	*p* = 0.519	*p* = 0.723	*p* = 0.503	*p* = 0.568	*p* = 0.621	*p* = 0.221	*p* = 0.884
	*R*^2^ = 0.062	*R*^2^ = 0.027	*R*^2^ = 0.039	*R*^2^ = 0.012	*R*^2^ = 0.042	*R*^2^ = 0.030	*R*^2^ = 0.023	*R*^2^ = 0.133	*R*^2^ = 0.002
Verrucomicrobiae	**7.9**	15	1.6	1	8.4	4.1	1.8	3.8	−6.1
	**(1.9, 14.0)**	(−0.3, 30.2)	(−1.1, 4.3)	(−0.5, 2.6)	(−0.6, 17.4)	(−0.3, 8.4)	(−4.6, 8.2)	(−2.9, 10.5)	(−21.6, 9.3)
	***p* = 0.015**	*p* = 0.053	*p* = 0.214	*p* = 0.178	*p* = 0.065	*p* = 0.063	*p* = 0.551	*p* = 0.241	*p* = 0.401
	***R***^**2**^ **= 0.429**	*R*^2^ = 0.299	*R*^2^ = 0.137	*R*^2^ = 0.159	*R*^2^ = 0.276	*R*^2^ = 0.281	*R*^2^ = 0.033	*R*^2^ = 0.123	*R*^2^ = 0.065

*In each of the models, the associations are reported as absolute difference (β), with 95% Confidence Intervals (CI), in immune system parameters levels by 1 unit increase in gut microbiota. In bold are reported the statistically significant results (p < 0.05). Per each of the models, the Coefficient of determination (R^2^) is also reported*.

**Table 5:** Associations from linear regression models between gut microbiota and alterations of adaptive immune system parameters measured in the spleen.

	**IL-17** **β (95% CI)**	**IFNg** **β (95% CI)**	**Myeloid cells** **β (95% CI)**	**CD45+** **β (95% CI)**	**CD11b+** **β (95% CI)**	**CD11c+** **β (95% CI)**	**F480+** **β (95% CI)**	**Ly6G** **β (95% CI)**
Actinobacteria	−0.4	−2.5	−7.3	−6.4	−5.2	−1.1	−1.8	−3.9
	(−14.5, 13.7)	(−18.6, 13.6)	(−21.0, 6.4)	(−17.1, 4.4)	(−13.9, 3.6)	(−2.6, 0.5)	(−4.9, 1.4)	(−9.7, 2.0)
	*p* = 0.947	*p* = 0.726	*p* = 0.267	*p* = 0.220	*p* = 0.220	*p* = 0.155	*p* = 0.240	*p* = 0.171
	*R*^2^ = 0.001	*R*^2^ = 0.019	*R*^2^ = 0.111	*R*^2^ = 0.134	*R*^2^ = 0.133	*R*^2^ = 0.175	*R*^2^ = 0.123	*R*^2^ = 0.163
Alphaproteobacteria	−0.9	−0.8	0.3	0.3	0.2	0.1	0	0.1
	(−3.0, 1.2)	(−3.3, 1.6)	(−2.1, 2.7)	(−1.7, 2.2)	(−1.4, 1.7)	(−0.2, 0.3)	(−0.5, 0.6)	(−0.9, 1.2)
	*p* = 0.349	*p* = 0.454	*p* = 0.769	*p* = 0.771	*p* = 0.826	*p* = 0.671	*p* = 0.877	*p* = 0.807
	*R*^2^ = 0.126	*R*^2^ = 0.082	*R*^2^ = 0.008	*R*^2^ = 0.008	*R*^2^ = 0.005	*R*^2^ = 0.017	*R*^2^ = 0.002	*R*^2^ = 0.006
Bacilli	0.1	−0.6	−0.5	−0.4	−0.3	0	−0.1	−0.2
	(−1.8, 2.0)	(−2.7, 1.6)	(−2.5, 1.6)	(−2.0, 1.2)	(−1.7, 1.0)	(−0.3, 0.2)	(−0.6, 0.3)	(−1.1, 0.7)
	*p* = 0.912	*p* = 0.547	*p* = 0.615	*p* = 0.602	*p* = 0.598	*p* = 0.688	*p* = 0.570	*p* = 0.588
	*R*^2^ = 0.002	*R*^2^ = 0.054	*R*^2^ = 0.024	*R*^2^ = 0.026	*R*^2^ = 0.026	*R*^2^ = 0.015	*R*^2^ = 0.030	*R*^2^ = 0.028
Bacteroidia	−0.1	−0.2	**0.2**	**0.2**	**0.1**	**0**	**0.1**	**0.1**
	(−0.3, 0.1)	(−0.4, 0.1)	**(0.0, 0.4)**	**(0.0, 0.3)**	**(0.0, 0.3)**	**(0.0, 0.0)**	**(0.0, 0.1)**	**(0.0, 0.2)**
	*p* = 0.362	*p* = 0.217	***p* = 0.020**	***p* = 0.015**	***p* = 0.015**	***p* = 0.005**	***p* = 0.021**	***p* = 0.011**
	*R*^2^ = 0.120	*R*^2^ = 0.208	***R***^**2**^ **= 0.402**	***R***^**2**^ **= 0.432**	***R***^**2**^ **= 0.429**	***R***^**2**^ **= 0.519**	***R***^**2**^ **= 0.399**	***R***^**2**^ **= 0.459**
Betaproteobacteria	−16.2	−18.6	14	11.8	9.5	1.9	3.1	6.2
	(−43.6, 11.2)	(−50.1, 12.9)	(−5.7, 33.6)	(−3.5, 27.2)	(−3.1, 22.0)	(−0.3, 4.1)	(−1.4, 7.7)	(−2.4, 14.8)
	*p* = 0.204	*p* = 0.206	*p* = 0.146	*p* = 0.118	*p* = 0.125	*p* = 0.082	*p* = 0.159	*p* = 0.140
	*R*^2^ = 0.219	*R*^2^ = 0.217	*R*^2^ = 0.182	*R*^2^ = 0.207	*R*^2^ = 0.200	*R*^2^ = 0.249	*R*^2^ = 0.172	*R*^2^ = 0.187
Clostridia	0.2	0.1	**−0.2**	**−0.2**	**−0.2**	**0**	**−0.1**	**−0.1**
	(−0.1, 0.4)	(−0.2, 0.4)	**(−0.4, −0.0)**	**(−0.4, −0.0)**	**(−0.3, −0.0)**	**(−0.1, −0.0)**	**(−0.1, −0.0)**	**(−0.2, −0.0)**
	*p* = 0.157	*p* = 0.410	***p* = 0.025**	***p* = 0.019**	***p* = 0.021**	***p* = 0.007**	***p* = 0.029**	***p* = 0.015**
	*R*^2^ = 0.264	*R*^2^ = 0.099	***R***^**2**^ **= 0.379**	***R***^**2**^ **= 0.405**	***R***^**2**^ **= 0.396**	***R***^**2**^ **= 0.493**	***R***^**2**^ **= 0.363**	***R***^**2**^ **= 0.429**
Deferribacteres	1.3	4.9	−6.6	−5.2	−4.5	−0.8	−1.6	−3
	(−10.4, 13.0)	(−7.9, 17.7)	(−17.9, 4.8)	(−14.2, 3.7)	(−11.7, 2.8)	(−2.1, 0.5)	(−4.2, 1.0)	(−7.9, 2.0)
	*p* = 0.802	*p* = 0.396	*p* = 0.228	*p* = 0.226	*p* = 0.204	*p* = 0.199	*p* = 0.204	*p* = 0.213
	*R*^2^ = 0.010	*R*^2^ = 0.105	*R*^2^ = 0.129	*R*^2^ = 0.130	*R*^2^ = 0.142	*R*^2^ = 0.145	*R*^2^ = 0.142	*R*^2^ = 0.137
Deltaproteobacteria	−1.5	0.5	0.1	0	−0.1	0	0	0
	(−3.2, 0.2)	(−2.0, 3.0)	(−2.0, 2.1)	(−1.6, 1.7)	(−1.4, 1.3)	(−0.2, 0.3)	(−0.5, 0.4)	(−0.9, 0.9)
	*p* = 0.083	*p* = 0.641	*p* = 0.945	*p* = 0.950	*p* = 0.933	*p* = 0.918	*p* = 0.879	*p* = 0.962
	*R*^2^ = 0.369	*R*^2^ = 0.033	*R*^2^ = 0.001	*R*^2^ = 0.000	*R*^2^ = 0.001	*R*^2^ = 0.001	*R*^2^ = 0.002	*R*^2^ = 0.000
Erysipelotrichia	−1.8	**9**	−3.9	−3.2	−2.6	−0.5	−1	−1.8
	(−9.9, 6.3)	**(4.0, 14.0)**	(−12.5, 4.7)	(−10.0, 3.6)	(−8.2, 2.9)	(−1.5, 0.5)	(−2.9, 1.0)	(−5.6, 2.0)
	*p* = 0.612	***p* = 0.004**	*p* = 0.336	*p* = 0.320	*p* = 0.321	*p* = 0.321	*p* = 0.301	*p* = 0.323
	*R*^2^ = 0.039	***R***^**2**^ **= 0.723**	*R*^2^ = 0.084	*R*^2^ = 0.090	*R*^2^ = 0.089	*R*^2^ = 0.089	*R*^2^ = 0.097	*R*^2^ = 0.089
Gammaproteobacteria	−15.7	−7.6	−0.1	−1.1	−0.8	−0.5	−0.1	−1.7
	(−60.9, 29.6)	(−61.7, 46.4)	(−26.9, 26.6)	(−22.4, 20.1)	(−18.0, 16.5)	(−3.6, 2.6)	(−6.3, 6.1)	(−13.4, 10.0)
	*p* = 0.440	*p* = 0.749	*p* = 0.991	*p* = 0.909	*p* = 0.926	*p* = 0.745	*p* = 0.975	*p* = 0.755
	*R*^2^ = 0.087	*R*^2^ = 0.016	*R*^2^ = 0.000	*R*^2^ = 0.001	*R*^2^ = 0.001	R2 = 0.010	*R*^2^ = 0.000	*R*^2^ = 0.009
Mollicutes	0.9	−0.1	−0.6	−0.5	−0.3	−0.1	−0.1	−0.2
	(−0.5, 2.3)	(−2.0, 1.8)	(−2.3, 1.1)	(−1.9, 0.9)	(−1.5, 0.8)	(−0.3, 0.1)	(−0.5, 0.3)	(−1.0, 0.5)
	*p* = 0.168	*p* = 0.908	*p* = 0.440	*p* = 0.442	*p* = 0.509	*p* = 0.349	*p* = 0.573	*p* = 0.505
	*R*^2^ = 0.252	*R*^2^ = 0.002	*R*^2^ = 0.055	*R*^2^ = 0.055	*R*^2^ = 0.041	*R*^2^ = 0.080	*R*^2^ = 0.030	*R*^2^ = 0.042
Multi−affiliation	0.3	**8**	−0.9	−0.7	−0.7	−0.1	−0.2	−0.4
	(−7.5, 8.1)	**(2.5, 13.5)**	(−4.7, 2.8)	(−3.7, 2.2)	(−3.1, 1.7)	(−0.6, 0.3)	(−1.1, 0.7)	(−2.0, 1.3)
	*p* = 0.921	***p* = 0.011**	*p* = 0.599	*p* = 0.593	*p* = 0.515	*p* = 0.469	*p* = 0.614	*p* = 0.614
	*R*^2^ = 0.002	***R***^**2**^ **= 0.629**	*R*^2^ = 0.026	*R*^2^ = 0.027	*R*^2^ = 0.039	*R*^2^ = 0.049	*R*^2^ = 0.024	*R*^2^ = 0.024
Negativicutes	−5.5	−5.8	**14.5**	10.8	9.1	1.3	**3.3**	5.5
	(−20.7, 9.6)	(−23.4, 11.8)	**(0.3, 28.7)**	(−0.8, 22.4)	(−0.1, 18.4)	(−0.4, 3.1)	**(0.0, 6.6)**	(−1.1, 12.1)
	*p* = 0.418	*p* = 0.464	***p* = 0.045**	*p* = 0.064	*p* = 0.053	*p* = 0.128	***p* = 0.048**	*p* = 0.094
	*R*^2^ = 0.096	*R*^2^ = 0.079	***R***^**2**^ **= 0.316**	*R*^2^ = 0.278	*R*^2^ = 0.300	*R*^2^ = 0.198	***R***^**2**^ **= 0.310**	*R*^2^ = 0.234
Verrucomicrobiae	**−1.6**	−1.1	**2.1**	**1.7**	**1.4**	**0.3**	**0.5**	**0.9**
	**(−3.2, −0.0)**	(−3.4, 1.1)	**(0.5, 3.8)**	**(0.5, 3.0)**	**(0.3, 2.4)**	**(0.1, 0.4)**	**(0.1, 0.9)**	**(0.2, 1.7)**
	***p* = 0.048**	*p* = 0.274	***p* = 0.015**	***p* = 0.012**	***p* = 0.016**	***p* = 0.008**	***p* = 0.024**	***p* = 0.015**
	***R***^**2**^ **= 0.451**	*R*^2^ = 0.167	***R***^**2**^ **= 0.431**	***R***^**2**^ **= 0.449**	***R***^**2**^ **= 0.422**	***R***^**2**^ **= 0.488**	***R***^**2**^ **= 0.385**	***R***^**2**^ **= 0.429**

*In each of the models, the associations are reported as absolute difference (β), with 95% Confidence Intervals (CI), in immune system parameters levels by 1 unit increase in gut microbiota. In bold are reported the statistically significant results (p < 0.05). Per each of the models, the Coefficient of determination (R^2^) is also reported*.

## Discussion

The results of our study in mice suggest that diet composition might have a pervasive role in co-regulating adaptive immunity and gut microbiota's profile in healthy obese subjects and in atherogenesis/inflammation in subjects with normal BMI. There has been recently a great focus on a particular subset of overweight and obese individuals having normal metabolic profile despite highly increased adipose mass (MHO = metabolic healthy obese; Karelis, 2008; Flegal et al., 2013). Individuals with adverse metabolic status despite a normal BMI have also been described (MSL = metabolic syndrome lean; Karelis, 2008; Flegal et al., 2013). It is currently unclear whether metabolic dysfunctions affects the higher morbidity and mortality observed in individuals with higher BMI: the concept of “benign obesity” has been challenged by some meta-analyses (Kramer et al., 2013) but not by others (Dhana et al., 2016), suggesting that MetS and not elevated BMI is an unequivocal risk factor for cardiovascular diseases (CVD). As it was previously reported, we confirmed that mice in a C57/BL6 genetic background fed a Paigen diet (PD) developed features of MetS, including hyperinsulinemia, hyperglycaemia, steatohepatitis, and inflammatory infiltration into the aorta, without increase in body weight (Getz and Reardon, 2006). Although, atherosclerosis is not observed without ApoE^−/−^ mutation in mice, this study reports for the first time a systemic activation of the immune system upon an atherogenic diet, with high tissue infiltration of myeloid cell subsets CD45+CD11b+CD11c, CD45+ F4/80+, CD45+ CD11b+Ly6G+ in the spleen, a massive lymphocyte infiltration in the aorta and in the liver, a significant increase in CD4+ and CD8+ positive cells in the aorta and in the hearts, paralleled by increased IL-17, IL-1, IFN-γ, and IL-4 levels in the aorta and heart. The elevated level of gene expression for IL-1α detected in the heart might indicate the activation of myeloid cell types, probably macrophages, which have been described as the main proinflammatory cell population in atherosclerotic plaques (Jonasson et al., 1986), and also play a crucial role in the development of heart failure (Heidt et al., 2014). In general, IL-1 critically orchestrates the inflammatory events that are considered building blocks for the formation atherosclerotic plaques, precursors and risk factor for CVD such as myocardial infarction (Van Tassell et al., 2013; Gallego-Colon et al., 2015; Taleb et al., 2015). In this study, mice in a C57/BL6 genetic background fed a high fat diet (HD) developed obesity, increased body weight and fatty liver without systemic inflammation and activation of the adaptive immune system. We took advantage of these two phenotypically characterized mice models of MSL and MHO, to scrutinize the composition of gut microbiota in the stool of these metabolic exceptions. High-throughput 16S targeted sequencing showed a dominion of Bacteroidia, Deltaproteobacteria and Verrucomicrobia and under-representation of Clostridia in MSL PD-fed mice. Generally, Proteobacteria and Verrucomicrobia are not abundant in the healthy gut, but abundant in the gut dysbiosis of patients with type 2 diabetes or with inflammatory bowel disease (IBD) (Larsen et al., 2010; Qin et al., 2012; Shin et al., 2015). More abundant bacteria such as Bacteroidiales and Clostridiales are more and less represented, respectively, in type 2 diabetes compared to obesity (Larsen et al., 2010; Qin et al., 2012). Bacteroidiales are associated to weight loss (Million et al., 2013). Our results are completely in line with the above reports and with the strong pro-inflammatory and pro-MetS role of PD compared to ND and HD. To our knowledge, a limited number of studies described clearly a role for gut microbiota in the onset of the MHO phenotype. It has been shown that separate cohorts of mice belonging to the same genetic background (C57/BL6) became either diabetic or resistant to diabetes and related metabolic dysfunctions despite being eating the same high-fat diet triggering obesity (Serino et al., 2012). The gut microbiota of the diabetes-resistant mice displayed a 20% decrease in the abundance of Firmicutes that were replaced by a parallel increase in Bacteriodetes (Serino et al., 2012). Moreover, the microbiota of diabetes-resistant mice presented with less bacteria of the *helicobacter* genus compared to the diabetic mice; instead, actinobacteria levels were unchanged (Serino et al., 2012). Our and these published studies suggest that the gut microbiota might reflect faithfully the metabolic phenotype irrespective of variability in the genetic background and diets of the host. Results of a recent study performed in the brown bear (*Ursus arctos*) are consistent with this (Sommer et al., 2016). The bear is a mammal accumulating enormous quantities of adipose fat in a seasonal manner (summer); by doing so, bears develop hyperlipidemia while maintaining metabolic health and being resistant to the development of atherosclerosis (Arinell et al., 2012). In fact, during summer season, the gut of bears harbored a different composition of microbiota than during winter season. In summer it was shown that gut microbiota was richer in Actinobacteria, Firmicutes, Proteobacteria, and poorer in Bacteroidetes (Sommer et al., 2016).

Several groups have provided data supporting a role for gut microbiota in the establishment of the MSL. If intestinal microbiota is suppressed in atherosclerosis-prone mice, an inhibition of dietary-choline-dependent atherosclerosis is observed (Wang et al., 2011). Patients with symptomatic atherosclerosis and normal body weight showed enrichment of the genus *Collinsella* of Actinobacteria in the gut (Karlsson et al., 2012). Generally, gut microbiota can affect atherosclerosis even in absence obesity or high fat feeding by different pathways: (i) infection activating the immune system and causing an inflammatory and proatherogenic response at distant sites; (ii) alteration of the levels of serum triglycerides and cholesterol, and of the metabolism of bile acids; (iii) dietary components (such as choline) and microbial metabolites [such as trimethylamine N-oxide (TMAO) generated from microbial metabolism of phosphatidylcholine, which is common in red meat and shellfish] lead to the production of both beneficial and harmful molecules (Jonsson and Backhed, 2017). For this reasons, gut microbiome is sometimes described as “endocrine” organ contributing to organism homeostasis [47].

Here, observed pro-inflammatory cytokines levels and tissue infiltrates correlating with decreased numbers of Clostridia corroborate previous findings on their regulatory functions. Clostridia strains presented in colon environment synergise to induce Tregs development [48, 49]. Tregs are fundamental to maintain mucosal homeostasis; therefore their insufficient development has pathological potential. Atherosclerosis develops upon stimulation of dendritic cells with oxidized low-density lipoproteins, the pathology is orchestrated by Th17 produced IL-17 [50], pro-autoimmune role of Th17 in atherosclerosis as well as association to HD induced chronic inflammation is well-described. Bacteroidetes has been associated to “healthy” non-obese homeostatic microbiome and with immunomodulation [51]. Interestingly addition of short chain fatty acids to the diet can result in Bacteroidetes abundance also during HD [52]. *Bacteroides fragilis* polysaccharide A (PSA) promotes T cells development [53], furthermore dysbiosis is frequently described as reduced Firmicutes/Bacteroidetes ratio, and this change was associated with IL-17 production and Th17 responses [54, 55].

Our result shows that increase proportion of Verrucomicrobiae correlates with higher percentage of myeloid cells markers. Interestingly, Roopchand et al. has shown that presence of *A. muciniphila* from Verrucomicrobiae together with presence of Bacteroidetes has significant role in protection to diet-induced obesity and metabolic dysbiosis in mice fed with HD [56]. Furthermore, in support to our data Ganesh et al. showed that presence of *A. muciniphila* increased levels of IL-17 in Salmonella infected mice [57].

It has been already demonstrated that diet shapes gut microbiome composition (De Filippo et al., 2010), and it is also now recognized that commensal microorganisms impact host gene expression not only in the gastrointestinal tract but also in other systems (Levy et al., 2015). Moreover, microbial cell components and secreted intermediate metabolites appear to be implicated in the response of the host to microbial colonization at the level of gene expression, which in turn reciprocally influence the disease progression. Noteworthy, both immunosuppressive drugs and probiotics affect the balance between microbiota and the immune system (Bartman et al., 2015). Particularly, probiotics supplementation have been shown to be effective in restoring and/or renovating the microbiota changes stimulating a number of health benefits, nevertheless whether modulation of gastrointestinal microbiota composition could have an effect on the amelioration of metabolic syndrome in obese or lean subjects, remains to be further investigated.

Supporting the link between metagenomics and immunogenomics, our data underline that understanding the reciprocal cross-talk between host immunity and microbiota will pave the way to the development of new therapeutic strategies against microbiome-driven common diseases, such as the metabolic syndrome.

## Author contributions

All authors made substantial contributions to the conception of the work; to the acquisition, analysis, or interpretation of data for the work; to the drafting the work and revising it critically for important intellectual content; and they finally approved the version to be published. All authors are accountable for all aspects of the work in ensuring that questions related to the accuracy or integrity of the work.

### Conflict of interest statement

The authors declare that the research was conducted in the absence of any commercial or financial relationships that could be construed as a potential conflict of interest. The reviewer PCS declared a shared affiliation, though no other collaboration, with the authors CS and MV to the handling Editor, who ensured that the process nevertheless met the standards of a fair and objective review.
